# The druggable transcription factor Fli-1 regulates T cell immunity and tolerance in graft-versus-host disease

**DOI:** 10.1172/JCI143950

**Published:** 2022-11-01

**Authors:** Steven D. Schutt, Yongxia Wu, Arjun Kharel, David Bastian, Hee-Jin Choi, Mohammed Hanief Sofi, Corey Mealer, Brianyell McDaniel Mims, Hung Nguyen, Chen Liu, Kris Helke, Weiguo Cui, Xian Zhang, Yaacov Ben-David, Xue-Zhong Yu

**Affiliations:** 1 Department of Microbiology and Immunology, Medical University of South Carolina (MUSC), Charleston, South Carolina, USA.; 2 Department of Microbiology and Immunology, Medical College of Wisconsin (MCW), Milwaukee, Wisconsin, USA.; 3 Department of Pathology, Yale School of Medicine, New Haven, Connecticut, USA.; 4 Department of Comparative Medicine,; 5 Department of Medicine at MUSC, Charleston, South Carolina, USA.; 6 Guizhou Medical University and the Key Laboratory of Chemistry for Natural Products of Guizhou Province and Chinese Academic of Sciences, Guiyang, China.; 7 Hollings Cancer Center, Medical University of South Carolina, Charleston, South Carolina, USA.; 8 The Cancer Center in MCW, Milwaukee, Wisconsin, USA.

**Keywords:** Immunology, Transplantation, Adaptive immunity, Bone marrow transplantation

## Abstract

Graft-versus-host disease (GVHD), manifesting as either acute (aGVHD) or chronic (cGVHD), presents significant life-threatening complications following allogeneic hematopoietic cell transplantation. Here, we investigated Friend virus leukemia integration 1 (Fli-1) in GVHD pathogenesis and validated Fli-1 as a therapeutic target. Using genetic approaches, we found that Fli-1 dynamically regulated different T cell subsets in allogeneic responses and pathogenicity in the development of aGVHD and cGVHD. Compared with homozygous *Fli1*-deficient or WT T cells, heterozygous *Fli1*-deficient T cells induced the mildest GVHD, as evidenced by the lowest Th1 and Th17 cell differentiation. Single-cell RNA-Seq analysis revealed that Fli-1 differentially regulated CD4^+^ and CD8^+^ T cell responses. Fli-1 promoted the transcription of Th1/Th17 pathways and T cell receptor–inducible (TCR-inducible) transcription factors in CD4^+^ T cells, while suppressing activation- and function-related gene pathways in CD8^+^ T cells. Importantly, a low dose of camptothecin, topotecan, or etoposide acted as a potent Fli-1 inhibitor and significantly attenuated GVHD severity, while preserving the graft-versus-leukemia (GVL) effect. This observation was extended to a xenograft model, in which GVHD was induced by human T cells. In conclusion, we provide evidence that Fli-1 plays a crucial role in alloreactive CD4^+^ T cell activation and differentiation and that targeting Fli-1 may be an attractive strategy for treating GVHD without compromising the GVL effect.

## Graphical abstract

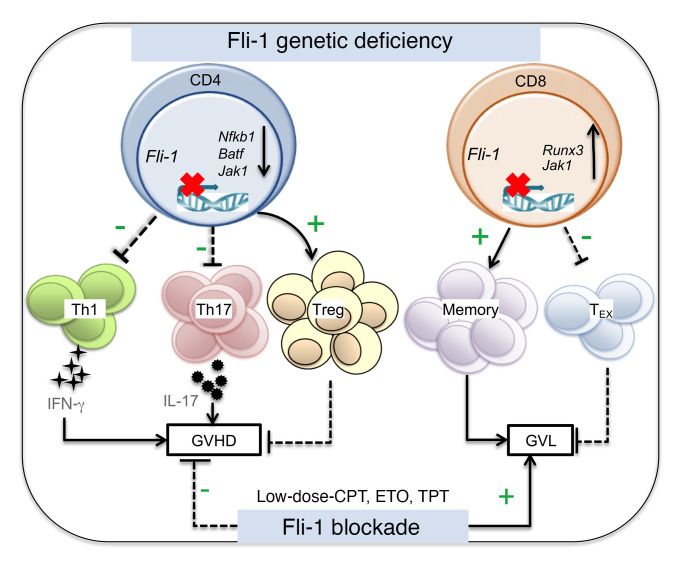

## Introduction

Currently, the most effective treatment in the clinic for hematological malignancies including leukemia, lymphoma, and myeloma is allogeneic hematopoietic cell transplantation (allo-HCT). Donor bone marrow (BM) or peripheral blood (PB) lymphocytes directly recognize and kill malignant cells within the host, termed the graft-versus-tumor (GVT) effect or graft-versus-leukemia (GVL) effect. A detrimental side effect of allo-HCT that occurs in 30%–70% of transplant recipients is chronic graft-versus-host disease (cGVHD). cGVHD is the primary cause of late-stage transplant-related morbidity and mortality despite available prophylactic strategies and treatments (1, 2).

Targeting T cell pathways remains a promising area of investigation in GVHD therapeutics. One potential T cell candidate target relevant to both leukemia and the immune response that has not, to our knowledge, been studied in GVHD pathogenesis is the transcription factor Friend virus leukemia integration 1 (Fli-1). Although Fli-1 has been relatively understudied specifically in primary lymphocytes, especially T cells, it was indeed previously demonstrated that retroviral overexpression of *Fli1* in T cell progenitor cells led to initiation of uncontrolled T cell proliferation and pre–T cell lymphoblastic lymphoma mediated by notch-1 receptor protein (NOTCH-1) mutations (3), and that T cells from germline heterozygous *Fli1*-deficient mice bearing the Fas mutation showed that Fli-1 was positively associated with the inflammatory factors CXCR3, IL-6, C16-ceramide, IL-17, and GM-CSF (4–8). These factors can also play important roles in GVHD pathogenesis (6, 9–13). A recent role for Fli-1 in regulating the CD8^+^ T cell response during infection and the antitumor response has also been identified, yet its role in primary CD4^+^ T cells remains largely elusive (14).

Cancer chemotherapeutics including camptothecin (CPT), the CPT analog topotecan (TPT), and etoposide (ETO) were shown to be potent Fli-1 protein inhibitors (15, 16)). In these studies, CPT impaired tumor growth in multiple erythroleukemia cell lines in vitro and in Friend murine leukemia virus–induced (F-MuLV-induced) erythroleukemia in vivo (15, 17), whereas TPT reduced lupus nephritis and inflammatory factors in human renal cells (16). Additionally, multiple cancer types that are targeted with allo-HCT such as acute myeloid leukemia (AML), lymphomas, and other hematopoietic malignancies have been shown to express high levels of *Fli1* mRNA, suggesting that targeting Fli-1 in these cancer types could be beneficial in reducing their growth (18).

Despite these previous findings, to date there has been no research to our knowledge that directly implicates Fli-1 as a link between immune tolerance and anti-leukemia immunity in the context of allo-HCT. Furthermore, whether Fli-1 plays an important role in the CD4^+^ T cell response has not to our knowledge been addressed until the current study. Here, we used a genetic strategy to target Fli-1 activity specifically on T cells and determined the role of Fli-1 in experimental GVHD models. We then used known pharmacological agents with strong Fli-1–inhibitory activity in preclinical allo-HCT mouse models of acute GVHD (aGVHD) and cGVHD, as well as in a humanized xenograft model of GVHD, and showed that decreasing the expression or activity of Fli-1 may be an important translational concept for reducing the pathogenesis of GVHD without impairing the GVL response.

## Results

### Characteristics of Fli1 conditional-KO mice.

The *Fli1^fl/fl^* mouse strain was previously shown to effectively reduce Fli-1 mRNA and protein levels via Cre-mediated recombination on the Tie2 (Tek) promoter (19). We verified the ability of the *CD4* promoter–based Cre/*loxP* system to mediate effective *Fli1* recombination in T cells. Using a PCR probe specific for the consensus *loxP* sites present near exons 3 and 4, where, as expected, we found complete deletion of the exon 4 *loxP* site in *Fli1^fl/fl^CD4*Cre^+^ T cells compared with *Fli1^fl/WT^CD4*Cre^–^ T cells, which contained both *loxP* sites, but no *CD4*Cre recombinase expression, indicating that effective cell-specific *Fli1* recombination occurred in the presence of *CD4*Cre (Supplemental Figure 1A; supplemental material available online with this article; https://doi.org/10.1172/JCI143950DS1). In this Cre/*loxP* system, the *Fli1* exon 4 *loxP* site is cleaved, while the exon 3 *loxP* site is maintained after recombination (19). Furthermore, we found significantly reduced *Fli1* mRNA levels in T cells from *Fli1^fl/WT^*Cre^+^ (*Fli1^Het^*) and *Fli1*^fl/fl^Cre^+^ (*Fli1^KO^*) mice compared with *Fli1^fl/WT^*Cre^–^ (*Fli1^WT^*) controls (Supplemental Figure 1B).

Notch-1 is a signaling component known to be essential for IL-2 production as well as GVHD development (20–23), and, because Fli-1 has already been shown to be positively associated with Notch-1^+^ mutations in pre–T cell lymphoblastic lymphoma (pre-TLL) (3), we investigated whether this phenomenon would also apply to murine activated primary T cells. Within 48 hours of polyclonal T cell activation, reduced Fli-1 activity was associated with a reduction in *Notch1* mRNA levels in both *Fli1^fl/WT^*Cre^+^ and *Fli1^fl/fl^*Cre^+^ T cells (Supplemental Figure 1B). Fli-1 is also a known regulator of Ship-1 levels in transformed erythroid cells (24), prompting us to examine the expression of this phosphatase, which revealed that *Fli1^fl/fl^*Cre^+^, but not *Fli1^fl/WT^*Cre^+^, T cells had significantly lower *Inpp5d* (aka Ship-1) mRNA levels than did *Fli1^WT/WT^* T cells (Supplemental Figure 1B). These quantitative reverse transcription PCR (qPCR-PCR) data indicated that genetic ablation of *Fli1* exons 3 and 4 caused loss of function and transcriptional activity of Fli-1, as previously described (19). Western blotting showed a moderate reduction in Fli-1 protein expression in resting and polyclonally activated T cells with *Fli1* exon 3 and exon 4 genetic deletion (Supplemental Figure 1, C and D). We found no significant differences between groups in baseline frequencies of effector, effector memory, central memory, naive T cells, or natural Tregs in the spleen (Supplemental Figure 1E).

### T cell–specific Fli-1 mediates cGVHD development.

**Figure 1:**
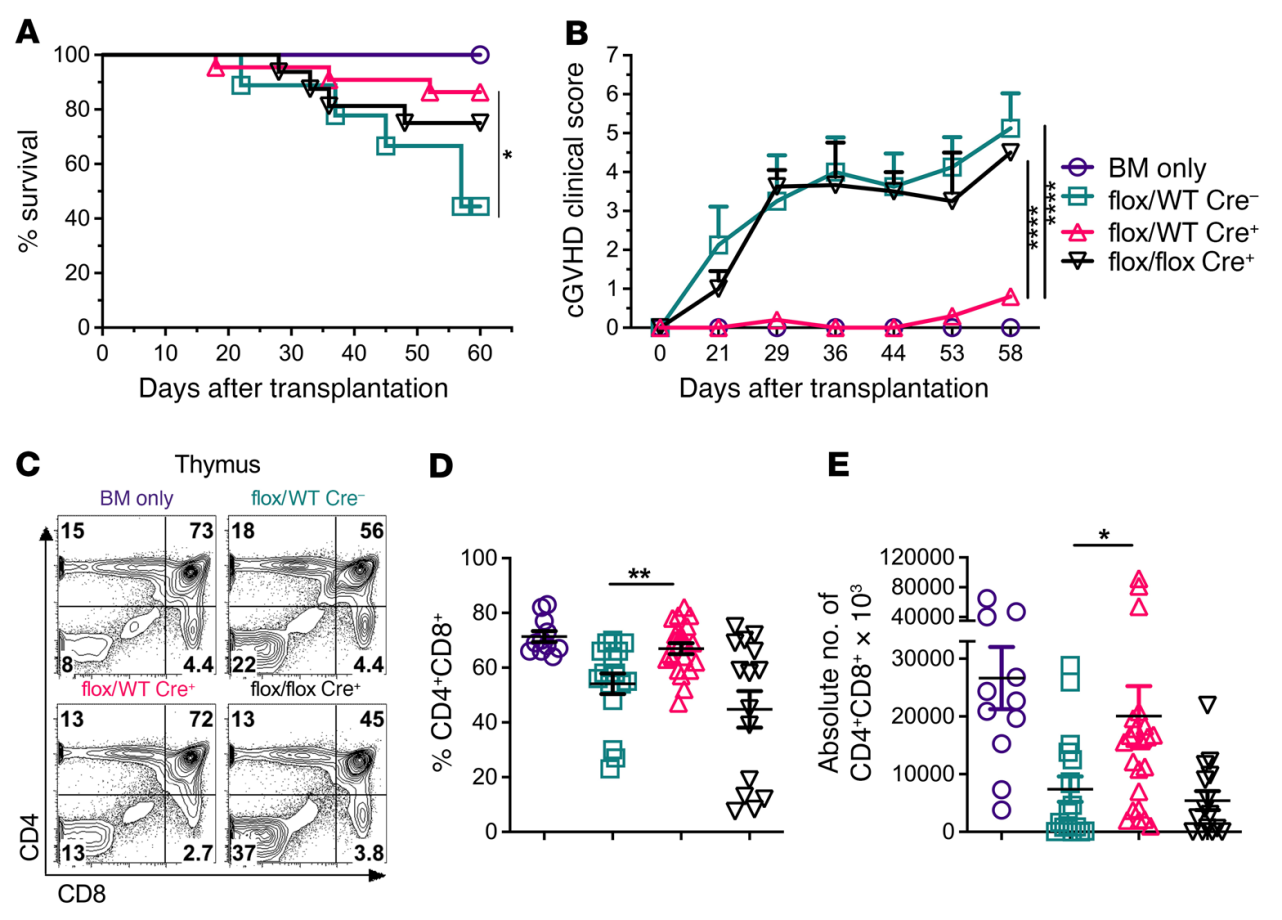
T cell–specific Fli-1 mediates cGVHD development. Lethally irradiated BALB/c mice were transplanted with 5 × 10^6^ TCD-BM and 0.5 × 10^6^ total splenocytes from *Fli1^WT^*, *Fli1^fl/WT^*, or *Fli1^fl/fl^* donors. Representative survival rates (**A**) and representative cGVHD clinical scores (**B**). Representative flow cytometry plots of CD4^+^CD8^+^ thymocytes from the experimental endpoint (days 45–60) (**C**), together with the cumulative frequency (**D**) and absolute number (**E**) of CD4^+^CD8^+^ thymocytes. Data represent 6 independent experiments (BM only, *n* = 12; *Fli1^WT^*, *n* = 17; *Fli1^fl/WT^*, *n* = 22; *Fli1^fl/fl^*, *n* = 15). Significance was determined using mixed-model tests for clinical scores, a log-rank (Mantel-Cox) test for survival data, and 1-way ANOVA for thymus data. *P < 0.05, **P < 0.01, and ****P < 0.0001.

To determine the role of Fli-1 in allo-HCT, we used a well-established preclinical cGVHD model. Under cGVHD conditions, donor marrow and splenocyte grafts from *Fli1^fl/WT^*Cre^+^ mice resulted in improved survival and a striking reduction in the cGVHD clinical score for recipient mice, but not for the recipients given *Fli1^fl/fl^*Cre^+^ or *Fli1^WT/WT^* grafts (Figure 1, A and B). We examined thymic reconstitution — a key marker of GVHD progression and severity — in these BM transplant recipients and found that recipients given *Fli1^fl/WT^*Cre^+^ grafts had superior CD4^+^CD8^+^ thymic reconstitution compared with those given either *Fli1^fl/fl^*Cre^+^ or *Fli1^WT/WT^* grafts (Figure 1, C–E).

Because CD4 and CD8 double-positive cells are generated from T cell progenitors that migrate from the BM (25), we tested whether *Fli1* deficiency played a role in the conversion of T cell progenitors to double-positive thymocytes by performing splenocyte and marrow chimeric BM transplantation (BMT). Here, recipients subjected to cGVHD conditions were transplanted with either *Fli1^WT/WT^*, *Fli1^fl/WT^*Cre^+^, or *Fli1^fl/fl^*Cre^+^ splenocytes (CD45.2), but each group received the same WT marrow bearing a congenic marker (CD45.1). Under these conditions, recipients given *Fli1^fl/fl^*Cre^+^ splenocytes and WT marrow had significantly increased frequencies of CD4^+^CD8^+^ thymocytes and a lower cGVHD clinical score compared with recipients given *Fli1^WT/WT^* splenocytes and WT marrow, yet recipients that received *Fli1^fl/WT^*Cre^+^ splenocytes and WT marrow grafts still had the lowest cGVHD clinical scores (Supplemental Figure 2A). Comparison of the matched splenocyte and marrow graft with the chimeric graft results suggested that T cell progenitors from *Fli1^fl/fl^*Cre^+^ marrow had a reduced ability to undergo normal CD4^+^CD8^+^ thymic reconstitution after BMT, as reflected by worse cGVHD clinical scores, whereas the recipients of *Fli1^fl/WT^*Cre^+^ grafts had improved thymic reconstitution, as reflected by low cGVHD clinical scores (Supplemental Figure 2, B and C). These data indicate that, while heterozygous *Fli1* mutation did not impact thymus development, homozygous loss of this transcription factor (TF) led to a significant reduction in normal thymic T cell frequencies, in agreement with previous observations that homozygous, but not heterozygous, ablation of Fli-1 impaired double-positive thymocytes (26). Thus, while a reduction of Fli-1 activity on mature T cells may be beneficial in reducing cGVHD, at least some Fli-1 activity may contribute to the conversion of T cell progenitors into CD4^+^CD8^+^ thymocytes after allo-BMT.

### Fli-1 dynamically inhibits Tregs and promotes T cell IFN-γ, IL-17A, and T follicular helper–like responses in vivo.

**Figure 2:**
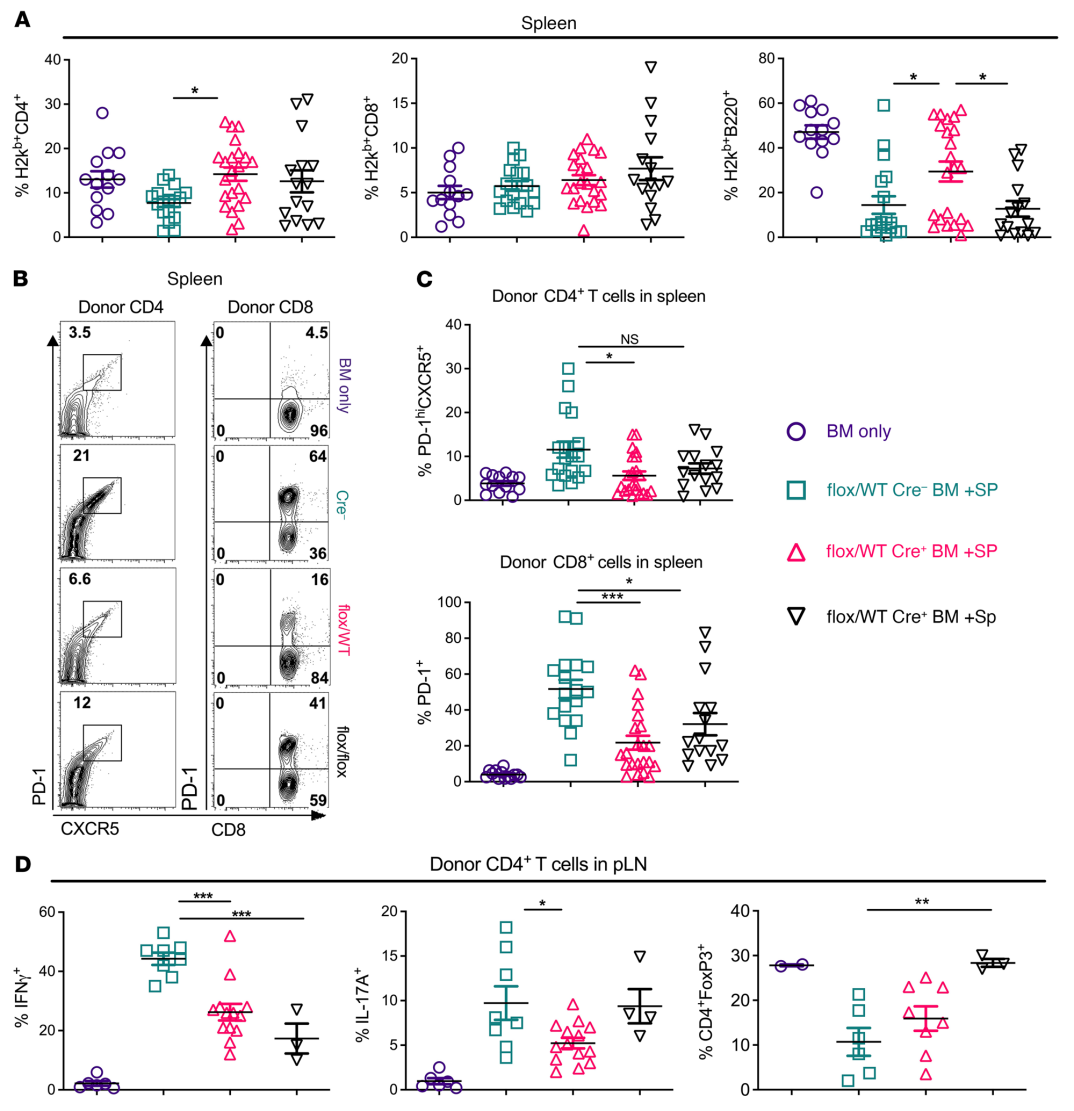
Fli-1 dynamically inhibits Tregs and promotes Th1, Th17, and Tfh responses in vivo. Lethally irradiated BALB/c mice were transplanted with 5 × 10^6^ TCD-BM and 0.5 × 10^6^ total splenocytes from *Fli1^WT^*, *Fli1^fl/WT^*, or *Fli1^fl/fl^* donors. Spleens and pLNs were collected from BM transplant recipients and analyzed at experimental endpoints via flow cytometry. Frequency of donor (H2K^b^) CD4^+^, CD8^+^, and B220^+^ cells from spleens (**A**). Representative flow cytometry plots (**B**) and cumulative frequencies of donor CD4^+^→PD-1^+^CXCR5^+^ or CD8^+^PD-1^+^ cell populations in recipient spleens (**C**). Cell isolates from pLNs of BM transplant recipients and frequencies of IFN-γ^+^, IL-17A^+^, and FoxP3^+^ cells from the donor CD4^+^ compartment (**D**). Data in **A**–**C** represent 6 independent experiments (BM only, *n* = 13; *Fli1^WT^*, *n* = 17; *Fli1^fl/WT^*, *n* = 22; *Fli1^fl/fl^*, *n* = 15), and data in **D** represent 3 independent experiments (BM only, *n* = 2-6; *Fli1^WT^*, *n* = 6–8; *Fli1^fl/WT^*, *n* = 6–13; *Fli1^fl/fl^*, *n* = 3–4). Data are shown as the mean ± SEM. Significance was determined by 1-way ANOVA with Tukey’s honest significant difference (HSD) post hoc analysis. *P < 0.05, **P < 0.01, and ***P < 0.001.

To further understand how Fli-1 regulates T cells to control cGVHD disease development, we examined different types of T cell subsets and T cell phenotypes within a secondary lymphoid organ (spleen) and in peripheral lymph nodes (pLNs) of mice at late time points after BMT. Mice that received matched *Fli1^fl/WT^*Cre^+^ splenocyte and marrow grafts had enhanced donor-derived splenic CD4^+^, but not CD8^+^, T cell reconstitution compared with mice given *Fli1^WT/WT^* grafts. Only recipients of *Fli1^fl/WT^*Cre^+^ grafts had significantly higher B cell reconstitution (Figure 2A). We consistently found lower frequencies of donor-derived splenic PD-1^+^CXCR5^+^–expressing CD4^+^ T cells, commonly referred to as T follicular helper (Tfh) cells, in recipients of either *Fli1^fl/WT^* or *Fli1^fl/fl^*Cre^+^ grafts compared with frequencies in recipients of *Fli-1^WT/WT^* grafts, although only the difference between *Fli1^fl/WT^* and *Fli1^WT/WT^* recipients was statistically significant. We also observed a similar phenomenon of reduced programmed cell death 1 (PD-1) expression on CD8^+^ T cells (Figure 2, B and C). These data suggest that Fli-1 may contribute to the differentiation of Tth cells and CD8^+^ T cell activation. Within pLNs, we found significant reductions in the frequencies of donor-derived CD4^+^ T cells that produced IFN-γ in mice that received *Fli1^fl/WT^*Cre^+^ or *Fli1^fl/fl^*Cre^+^ grafts, but only *Fli1^fl/WT^*Cre^+^ T cells had significantly reduced frequencies of CD4^+^IL-17A^+^ T cells (Figure 2D). Frequencies of donor-derived CD4^+^FoxP3^+^ Tregs were also increased in the recipients of *Fli1^fl/WT^*Cre^+^ or *Fli1^fl/fl^*Cre^+^ grafts, but only significantly in the *Fli1^fl/fl^*Cre^+^ group compared with the *Fli1^WT/WT^* group (Figure 2D).

Using the chimeric model that donor peripheral T cells can be distinguished with BM-derived T cells from WT Ly5.1^+^ congenic donors, we found that, compared with the *Fli1^WT^* group, the marrow-derived cells in the recipients of *Fli1^fl/WT^*Cre^+^ grafts also had a significant reduction in the PD-1^+^CXCR5^+^ Tfh cell–like population, along with a significant trend toward increased PD-1^+^CXCR5^+^ cells that coexpressed CD4^+^FoxP3^+^, also known as T follicular regulatory–like (Tfr-like) cells (Supplemental Figure 2D). In both spleen and LNs, the recipients of *Fli1^fl/WT^*Cre^+^ T cells and WT marrow had significantly reduced frequencies of CD4^+^IL-17A^+^ T cells compared with recipients of *Fli1^WT/WT^* grafts. Frequencies of CD4^+^IFN-γ^+^ T cells were also reduced in the spleens and LNs of mice that received *Fli1^fl/WT^*Cre^+^ grafts, although the reductions were restricted to marrow-derived T cells. The recipients of *Fli1^fl/WT^*Cre^+^ grafts showed a significant trend toward increased CD4^+^FoxP3^+^ Tregs within the marrow-derived compartment of the pLNs compared with the *Fli1^WT/WT^* group (Supplemental Figure 2, E–G). Taken together, these findings demonstrate that Fli-1 played an important and dynamic role in regulating the presence of pathogenic CD4^+^IFN-γ ^+^ Th1, CD4^+^IL-17A^+^ Th17, PD-1^+^CXCR5^+^ Tfh, and protective CD4^+^FoxP3^+^ Treg subsets in lymphoid organs of the recipient mice with cGVHD.

### Fli-1 inhibits antigen-specific induced Treg function while promoting IL-2 secretion and Th17 differentiation in vitro.

Little is known about the potential roles of Fli-1 in normal primary T cell biology, thus, we decided to create a T cell receptor–transgenic (TCR-Tg) mouse strain paired with our *Fli1^fl/fl^*CD4*Cre* strain to study the role of this TF in antigen-specific T cell responses. CD4^+^ T cells from these TCR-Tg mice are only able to respond to HY-peptide (27). To study the effects of Fli-1 on the antigen-specific T cell response, we polarized CD4^+^ T cells with HY-peptide and different cytokine cocktails to induce Th1, Th17, or induced Treg (iTreg) differentiation. Strikingly, we found that both *Fli1^fl/WT^*Cre^+^ and *Fli1^fl/fl^*Cre^+^ TCR-Tg cells had enhanced iTreg (CD25^+^FoxP3^+^) differentiation and expression of iTreg functional molecules (CD25, CD39, CD73, and NRP-1) compared with TCRtg *Fli1^WT/WT^* iTregs (Supplemental Figure 3, A and B). Further, both *Fli1^fl/WT^*Cre^+^ and *Fli1^fl/fl^*Cre^+^ TCR-Tg cells had a significant reduction in IL-17A production compared with *Fli1^WT/WT^* cells under Th17-polarizing conditions (Supplemental Figure 3, C and D). To evaluate the impact of Fli-1 on T cell growth and survival, we tested the abundance of IL-2 cytokines secreted into culture media from Th17- and Th1-polarizing cultures. We found that culture supernatants from both *Fli1^fl/WT^*Cre^+^ and *Fli1^fl/fl^*Cre^+^ cultures had significantly reduced levels of IL-2, suggesting that Fli-1 regulated antigen-specific T cell IL-2 production (Supplemental Figure 3, E and F). Together, these results suggest that Fli-1 contributed to the enhancement of Th17 polarization, while suppressing iTreg differentiation.

### Fli-1 regulates T cell pathogenicity in aGVHD.

**Figure 3:**
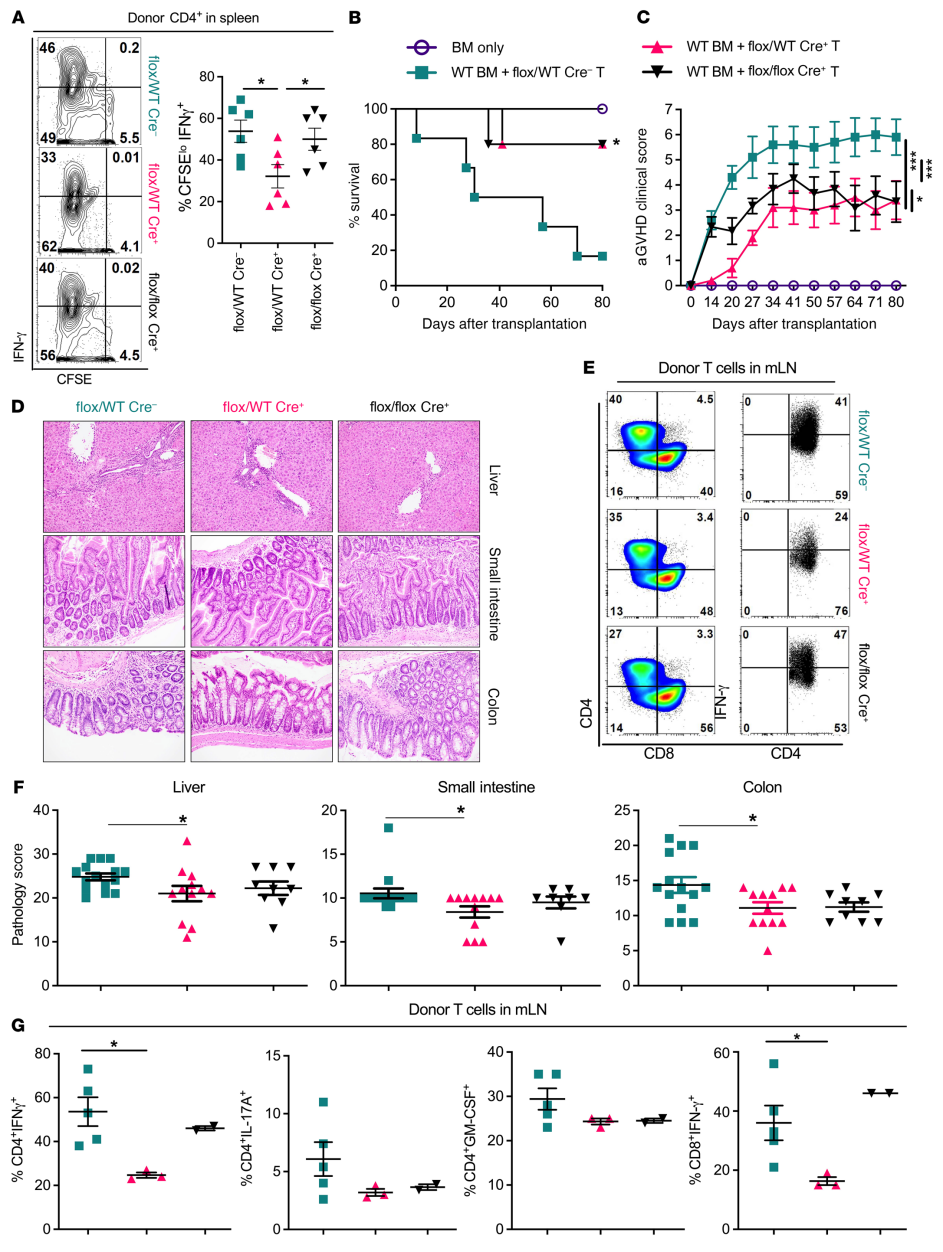
Fli-1 regulates T cell pathogenicity in aGVHD. Purified T cells from spleens and LNs of *Fli1^WT/WT^*, *Fli1^fl/WT^*, and *Fli1^fl/fl^* mice were CFSE labeled and infused into lethally irradiated BALB/c mice at 2 × 10^6^ cells per mouse. Day-4 representative flow cytometry plots and cumulative frequencies of proliferated (CFSE^lo^) donor^+^CD4^+^ cells producing IFN-γ (**A**) (*Fli1^WT^*, *n* = 6; *Fli1^fl/WT^*, *n* = 6; *Fli1^fl/fl^*, *n* = 6). Lethally irradiated BALB/c mice were transplanted with 5 × 10^6^ TCD-BM from CD45.1 B6 donors supplemented or not with 0.5 × 10^6^ purified total T cells from spleens and LNs of *Fli1^WT/WT^*, *Fli1^fl/WT^*, and *Fli1^fl/fl^* donors. aGVHD representative survival rates (**B**) and representative aGVHD clinical scores (**C**) (BM only, *n* = 7; *Fli1^WT^*, *n* = 17; *Fli1^fl/WT^*, *n* = 15; *Fli1^fl/fl^*, *n* = 15). On day 14 after BMT, the indicated tissues sections were H&E stained for pathologic scoring (**D**). mLNs were analyzed for donor T cell populations producing IFN-γ, IL-17A, or GM-CSF. Representative flow cytometry plots display IFN-γ–producing T cells in mLNs (**E**), and cumulative pathology scores are shown (**F**) (*Fli1^WT^*, *n* = 15; *Fli1^fl/WT^*, *n* = 12; *Fli1^fl/f^*,*^l^* *n* = 9). Frequencies of each indicated donor T cell population in mLNs (**G**) (*Fli1^WT^*, *n* = 5; *Fli1^fl/WT^*, *n* = 3; *Fli1^fl/fl^*, *n* = 2). Data in **A**–**F** represent 2–3 independent experiments. Data in **G** were collected from 1 set of mice belonging to 3 independent experiments. Significance was determined using mixed-model tests for clinical scores, a log-rank test for survival data, and 1-way ANOVA for all other data. *P < 0.05 and ***P < 0.001.

CD4^+^IFN-γ^+^ and/or IL-17A^+^ T cells play critical roles in aGVHD pathogenesis, which prompted us to determine whether Fli-1 can also contribute to aGVHD development. First, we examined early T cell activation and proliferation using an in vivo mixed lymphocyte reaction (MLR) model and found that donor *Fli1^fl/WT^*Cre^+^ CD4^+^ cells produced significantly lower levels of IFN-γ compared with both *Fli1^WT/WT^* and *Fli1^fl/fl^*Cre^+^ CD4^+^ cells (Figure 3A). We used the donor splenocyte and BM chimera strategy described above to determine the role of Fli-1 in aGVHD. Consistently, we found that recipients of either *Fli1^fl/WT^*Cre^+^ or *Fli1^fl/fl^*Cre^+^ grafts had significantly increased survival rates and reduced aGVHD clinical scores compared with recipients of *Fli1^WT/WT^* grafts (Figure 3, B and C). Among all 3 experimental groups, the recipients of *Fli1^fl/WT^*Cre^+^ grafts had the lowest aGVHD clinical scores and pathological damage in the liver, small intestine, and colon (Figure 3, D and F). Consistently, we found that T cells derived from *Fli1^fl/WT^*Cre^+^ donor grafts produced lower intracellular levels of IFN-γ in T cells than did *Fli1^WT/WT^* and *Fli1^fl/fl^*Cre^+^ donor T cells in recipient mesenteric LNs (mLNs) (Figure 3, E and G), a lymphoid organ that can closely reflect gut T cell migration and activation (28).

Indeed, alloreactive T cells are highly implicated in causing or exacerbating gut damage during GVHD (29). To extend our study beyond allogeneic responses, we used the classical syngeneic T cell transfer model of colitis to determine whether Fli-1 contributes to T cell–mediated gut damage. In support of our aGVHD findings, we observed that both *Fli1^fl/WT^*Cre^+^ and *Fli1^fl/fl^*Cre^+^ naive CD4^+^ T cells had a reduced ability to induce colitis, in which *Fli1^fl/WT^*Cre^+^ CD4^+^ T cells showed the least pathogenicity in colitis development (Supplemental Figure 4A). In addition, mice given *Fli1^fl/WT^*Cre^+^ or *Fli1^fl/fl^*Cre^+^ CD4^+^ T cells had reduced pathological damage in the colon compared with mice given *Fli1^WT/WT^* T cells (Supplemental Figure 4, B and C). Cumulatively, these data suggest that Fli-1 dynamically contributes to IFN-γ^+^–producing T cells during aGVHD development and that Fli-1 may be an important regulator of T cell pathogenicity in gut damage.

### Fli-1 contributes to the regulation of genes involved in Treg and effector T cell development and function.

To expand beyond the few target genes already known to be either positively or negatively regulated by Fli-1, we isolated purified T cells from the spleens of aGVHD mice transplanted with either *Fli1^WT/WT^*, *Fli1^fl/WT^*Cre^+^, or *Fli1^fl/fl^*Cre^+^ grafts and performed next-generation RNA-Seq during the cells’ peak expansion phase (day 14 after BMT). Consistently, we found that reduced Fli-1 activity was associated with a significant reduction in aGVHD clinical scores (Supplemental Figure 5A). RNA-Seq revealed multiple significantly downregulated and upregulated genes among each of the 3 genotypes tested. When comparing *Fli1^fl/WT^*Cre^+^ and *Fli1^fl/fl^*Cre^+^ T cells, the most significantly upregulated genes were also associated with antiinflammatory properties (e.g., *Foxp3, Dnase1L3, Lgals3* [galectin-3]) (30, 31), and the downregulated genes were associated with proinflammatory pathways (e.g., *Egr1*, *Crtam*, *Gpr18*) (32–34) (Supplemental Figure 5B). The most significantly upregulated genes in *Fli1^fl/WT^*Cre^+^ T cells compared with WT T cells were associated with antiinflammatory properties (e.g., *Foxp3, Zfp36l2*, *Tsc22d3* [aka GILZ]) (35), whereas the most significantly downregulated genes were related to effector T cell differentiation and function (e.g., *Ifng, Il21*, *Sema7a*) (Supplemental Figure 5C), suggesting together with our other in vitro and in vivo data that Fli-1 may be playing an important role in Treg development, while also being able to mediate pathogenic effector T cells. Comparing *Fli1^fl/fl^*Cre^+^ T cells with WT T cells revealed a mixed proinflammatory and antiinflammatory phenotype, in which some of the most significantly upregulated genes included *Ccr6*, *Pdcd1*, *Eomes*, and the most downregulated genes included *Tgfbi*, *IL6R*, *C1qa*, *C1qc*, and *Gzma* (Supplemental Figure 5D). Genes that are involved in Treg differentiation and function, including *Foxp3*, *Cd36* (36), *Tgfβ1*, *Il10ra* (37), and *Inpp5d* (aka SHIP-1) were confirmed with qRT-PCR (Supplemental Figure 5E). Genes related to effector T cell structure and function — *Ifng*, *Il21*, *Fas*, *Gpr18* (34), and *Sema7a* (38) — were also confirmed via qRT-PCR (Supplemental Figure 5F). On the basis of these RNA-Seq expression data, upstream regulator analysis via Ingenuity Pathway Analysis (IPA) also predicted significant differences in SIRT-1, NR4A1 (aka NUR77), IRF7, BCL6, TP53, and TCF7L2 pathways between *Fli1^fl/WT^*Cre^+^ and *Fli1^fl/fl^*Cre^+^ T cells, as well as between *Fli1^WT/WT^* T cells (Supplemental Figure 6), suggesting that these pathways could be the candidates underlying the significant differences observed in gene expression among the tested genotypes.

### Single-cell RNA-Seq analysis revealed discriminatory gene regulation in CD4^+^ versus CD8^+^ T cells by Fli-1.

**Figure 4:**
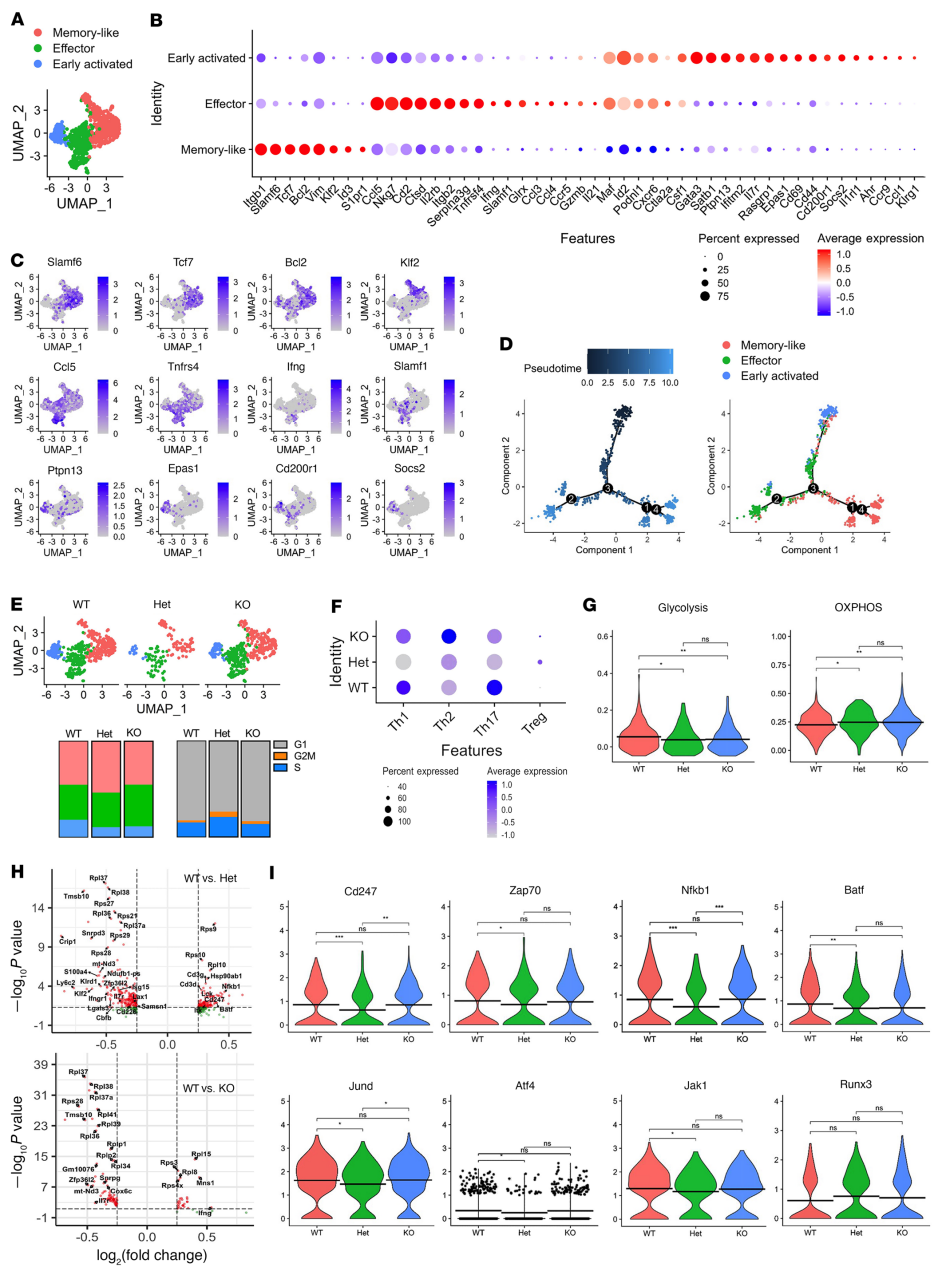
Fli-1 regulates gene transcription involved in the differentiation and function of CD4^+^ T cells. (**A**) Integrated UMAP showing 3 major CD4^+^ T cell clusters among donor T cells isolated from the spleens of BALB/c recipient mice that were transplanted with BM (*Rag1^–/–^*) and T cells from *Fli1^fl/WT^*Cre^–^ (*Fli1^WT^*), *Fli1^fl/WT^*Cre^+^ (*Fli1^Het^*), or *Fli1^fl/fl^*Cre^+^ (*Fli1^KO^*) donor mice on day 14. (**B**) Expression of cell-defining features across all cell types. Color intensity is proportional to the average gene expression in the indicated cell clusters. The size of the circles is proportional to the percentage of cells expressing the indicated genes. (**C**) mRNA expression of the indicated genes projected onto the UMAP in 3 cell subpopulations. (**D**) Single-cell trajectory of total CD4^+^ T cell subsets based on pseudotime (left) and cell type (right). (**E**) Integrated UMAP shows *Fli1^WT^*, *Fli1^Het^*, and *Fli1^KO^* CD4^+^ T cell clusters separately. Histogram shows the frequency of each cell cluster (left) and the frequency of cells in each cell-cycle phase (right) in *Fli1^WT^*, *Fli1^Het^*, and *Fli1^KO^* CD4^+^ T cells. (**F**) Dot plot shows Th1, Th2, Th17, and Treg gene module scores in early activated cells. (**G**) Violin plots indicate glycolysis and OXPHOS gene module scores for CD4^+^ T cells. (**H**) Volcano plots present the most DEGs between *Fli1^WT^* versus *Fli1^Het^* (top) and *Fli1^WT^* versus *Fli1^KO^* (bottom) CD4^+^ T cells. (**I**) Violin plots indicate the expression of the indicated genes in CD4^+^ T cells. Significance was determined by 1-way ANOVA. *P < 0.05, **P < 0.01, and ***P < 0.001.

To further understand how Fli-1 regulates T cell gene transcription and heterogeneity, we performed single-cell RNA-Seq (scRNA-Seq) analysis of donor T cells, including *Fli1^fl/WT^*Cre^+^ (*Fli1^WT^*), *Fli1^fl/WT^*Cre^+^ (*Fli1^Het^*), and *Fli1^fl/fl^*Cre^+^ (*Fli1^KO^*) T cells, isolated from recipient mouse spleens. An unbiased integrative analysis across all 3 genotypes after regression for potential artifacts using the Seurat platform resulted in 6,501 cells grouped into 9 subpopulations, in which we recognized cluster 3 for CD4^+^ T cells and clusters 1 and 6 for CD8^+^ T cells (Supplemental Figure 7A). The CD4^+^ and CD8^+^ T cells were further clustered on the basis of differential expression of genes and visualized using uniform manifold approximation and projection (UMAP) (Figure 4A and Supplemental Figure 7B). Clustering analysis revealed 3 major subpopulations defined according to the most salient identified cell markers: early activated, effector, and memory-like in both CD4^+^ and CD8^+^ T cells (Figure 4B and Supplemental Figure 7C).

In CD4^+^ T cells, early activated cells were identified by the expression of the TFs *Gata3*, *Satb1*, and *Epas1*; the activation markers *Ptpn13*, *Cd69*, *Cd44*, *Il1rl1*, and *Klrg1*; and the negative regulators *Rasgrp1, Cd200r1*, *Socs2*, and *Ahr* (Figure 4, B and C) (39–41). Activation markers, including *Maf*, *Id2*, *Cxcr6*, *Csf1*, were coexpressed by early activated and effector T cells. Effector T cells were further defined by the expression of the chemokines *Ccl5*, *Ccl3*, and *Ccl4*; the surface molecules *Nkg7*, *Cd2*, *Il2rb*, *Tnfrsf4*, and *Slamf1*; and the cytokines *Ifng*, *Gzmb*, and *Il21*. Memory-like T cells had the highest expression of the TFs *Tcf7*, *Bcl2*, *Klf2*, and *Id3* and of the immune receptors *Slamf6* and *S1pr1* (42, 43). Consistently, the Monocle algorithm predicted a differentiation trajectory with 1 major branch point, in which early activated CD4^+^ T cells could form both effector and memory-like cells, further confirming the linage relationship among 3 subsets (Figure 4D).

Loss of Fli-1 did not alter the subset distribution or the number of CD4^+^ T cell clusters, but rather increased the frequencies of memory-like cells relative to early activated cells in *Fli1^Het^* T cells compared with *Fli1^WT^* or *Fli1^KO^* CD4^+^ T cells (Figure 4E). Interestingly, cell-cycle analysis revealed an increased frequency of G_2_M- and S-phase cells in the *Fli1^Het^* CD4^+^ T cells (Figure 4E). We conducted a gene set enrichment analysis (GSEA) to examine the potential of early activated T cells to differentiate into Th1, Th2, Th17, or Treg subsets (Figure 4F). When compared with WT controls, both *Fli1^Het^* and *Fli1^KO^* CD4^+^ T cells exhibited decreased Th1 and Th17 gene module scores, whereas *Fli1^Het^* CD4^+^ T cells shad the lowest module scores (Figure 4F). *Fli1^Het^* and *Fli1^KO^* CD4^+^ T cells had higher Treg gene module scores, and *Fli1^KO^* T cells also had an increased Th2 module score. Lower glycolysis but higher oxidative phosphorylation (OXPHOS) gene module scores were obtained in *Fli1^Het^* and *Fli1^KO^* CD4^+^ T cells, suggesting that Fli-1 may regulate gene pathways related to cellular metabolism (Figure 4G). In addition, compared with *Fli1^WT^* controls, *Fli1^Het^* CD4^+^ T cells showed downregulation of TCR pathway genes, including *Cd3g*, *Cd3d*, *Lck*, *Cd247*, *Zap70*, and *Itk*, and lower expression of TCR-induced genes, including *Nfkb1*, *Batf*, *Jund*, *Atf4*, and *Jak1* (43–45). In contrast, *Fli1^Het^* CD4^+^ T cells showed upregulation of genes such as *Zfp36l2*, *Lgals3*, *Il7r*, *Lax1*, *Ifngr1*, and *Cd226*, which are involved in negative regulation of the immune effector process and among which *Zfp36l2* and *Lgals3* were also elevated in *Fli1^Het^* T cells in our bulk RNA-Seq data (Figure 4, H and I, and Supplemental Figure 5C) (46–49). In contrast to *Fli1^Het^* cells, fewer differentially expressed genes (DEGs) were observed when comparing *Fli1^KO^* with WT CD4^+^ T cells, among which *Ifng* was downregulated, while *Zfp36l2* and *Il7r* were upregulated in. We observed little difference in the expression of TCR downstream TF genes, with the exception of *Baft*, which was downregulated in *Fli1^KO^* CD4^+^ T cells (Figure 4, H and I). In summary, Fli-1 deficiency in CD4^+^ T cells modulates gene transcription involved in T cell differentiation and metabolism. Fli-1 heterozygous deficiency in CD4^+^ T cells modified the composition of early activated versus memory-like cells and showed additional impact on the transcription of TCR pathway and TCR downstream TF genes.

On the other hand, 3 clusters in CD8^+^ T cells were identified as early activated (*Ltb*, *Cxcr6*, *Id2*, *Ly6a*, *Rbpj*, *Plac8*, *Emb*, *Cxcr3*, and *Cd69*), effector (*Gzma*, *Gzmb*, *Ifng*, *Prf1*, *Ccl3*, *Ccl4*, *Pdcd1*, *Lag3*, *Havcr2*, and *Eomes*), and memory-like (*Klf2*, *Vim*, *S100a4*, *Lgals1*, *S1pr1*, and *Ly6c2*) (Supplemental Figure 7, C and D) (50). The differentiation trajectory showing that early activated CD8^+^ T cells could form both effector and memory cells further confirmed the cell type (Supplemental Figure 7E). *Fli1^Het^* CD8^+^ T cells had a similar cluster composition, whereas *Fli1^KO^* CD8^+^ T cells likely had decreased effector but increased memory-like cells compared with *Fli1^WT^* controls (Supplemental Figure 7F). Similar to observations in CD4^+^ T cells, increased frequencies of G_2_M-phase cells were also observed in *Fli1^Het^* CD8^+^ T cells (Supplemental Figure 7F). Both *Fli1^Het^* and *Fli1^KO^* CD8^+^ T cells had increased activation but decreased exhaustion gene module scores (Supplemental Figure 7G). *Fli1^Het^* CD8^+^ T cells had increased effector but reduced memory pathway gene enrichment compared with *Fli1^WT^* control CD8^+^ T cells, whereas *Fli1^KO^* CD8^+^ T cells showed an opposite trend. Consistent with the observation in CD4^+^ T cells, both *Fli1^Het^* and *Fli1^KO^* CD8^+^ T cells had increased OXPHOS, and *Fli1^KO^* CD8^+^ T cells showed reduced enrichment of glycolysis genes (Supplemental Figure 7H). Fli-1 deficiency in CD8^+^ T cells had little effect on TCR pathway and TCR downstream TF genes, and even increased the expression of *Jak1* (Supplemental Figure 7J). The expression of *Runx3*, a TF critical for cytotoxic T lymphocyte (CTL) program initiation and memory formation (51, 52), was increased in *Fli1^KO^* CD8^+^, but not CD4^+^, T cells (Supplemental Figure 7J and Figure 4I). Thus, Fli-1 deficiency in CD8^+^ T cells increased gene transcription for CD8^+^ T cell activation, function, and OXPHOS metabolism, and Fli-1 may regulate the CD4^+^ versus the CD8^+^ T cell response differentially at the transcriptional level.

### CPT, ETO, and TPT target Fli-1 and ameliorate cGVHD.

To determine whether inhibiting Fli-1 could be a potential translational strategy for targeting aberrant T cell activation and GVHD, we used a currently available pharmacological agent, CPT, which has been shown previously to potently inhibit Fli-1 (15). We confirmed that CPT could reduce Fli-1 protein expression in the murine T cell leukemia line EL4 (Supplemental Figure 8A), which was associated with reduced cell growth and increased apoptosis (Supplemental Figure 8, B and C). To determine the extent of specificity of low-dose CPT for Fli-1 versus topoisomerase I inhibition, we performed a topoisomerase I enzymatic activity assay and found that low-dose CPT did not significantly hinder the ability of topoisomerase I to relax supercoiled DNA — the primary function of this enzyme — even after a 48-hour incubation of activated T cells with low-dose CPT (Supplemental Figure 8D). We then tested the impact of low-dose CPT on primary murine polyclonally activated T cells in vitro and found that CPT reduced Fli-1 expression (Supplemental Figure 1D) and T cell proliferation but preserved IFN-γ production (Supplemental Figure 8, E and F). CPT treatment of purified polyclonally stimulated WT T cells or *Fli1^fl/fl^*Cre^+^ T cells revealed that a low concentration of CPT had a major effect on WT T cells via decreased IFN-γ^+^ and Ki-67^+^ frequencies, and there were modest, nonspecific effects of CPT against Fli-1 when cultured with *Fli1^fl/fl^*Cre^+^ T cells (Supplemental Figure 9A). In addition, CPT treatment suppressed activated effector T cells, while sparing Tregs when stimulated with allogeneic antigen-presenting cells (APCs) in vitro (Supplemental Figure 9, B and C).

**Figure 5:**
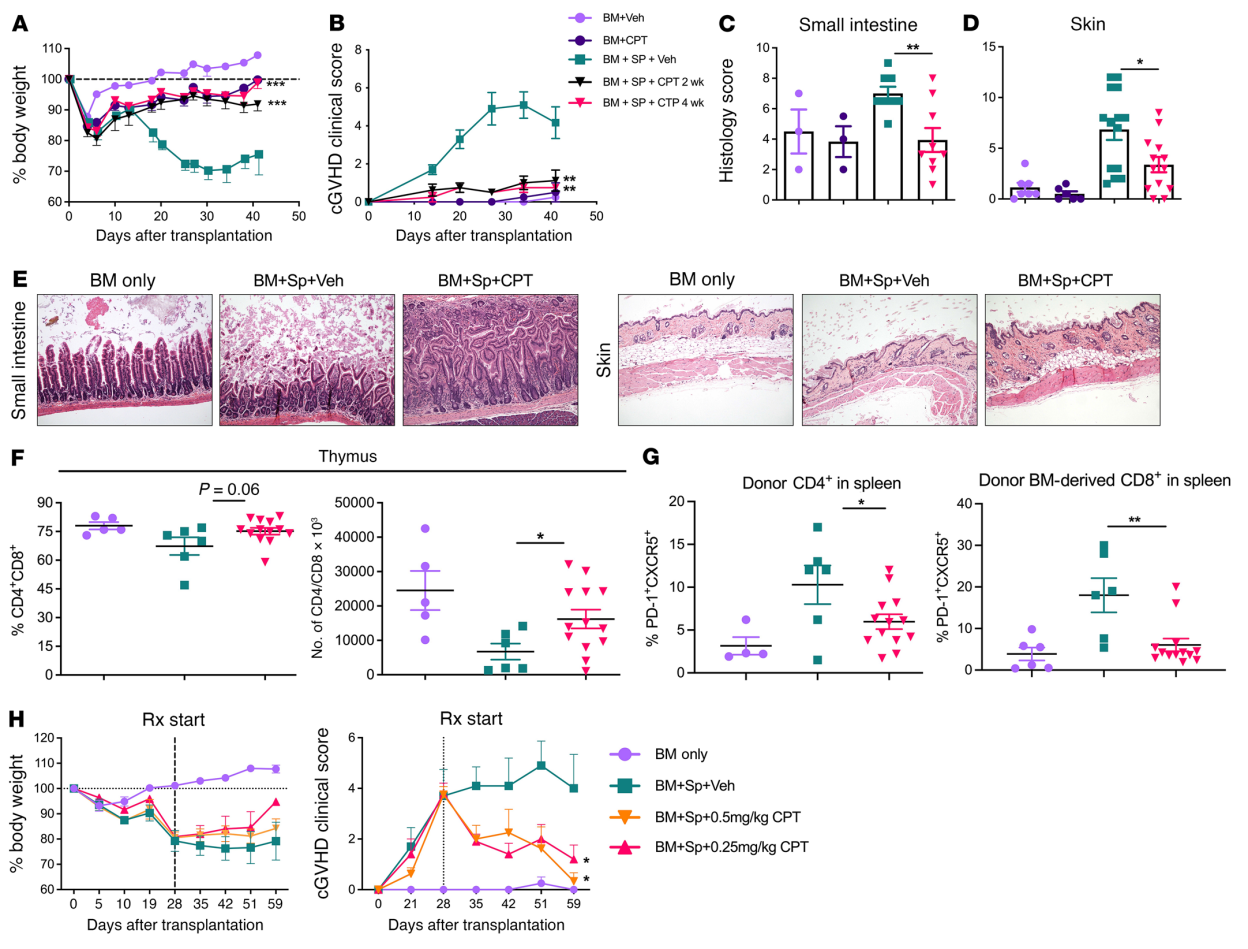
Low-dose CPT prevents and reverses cGVHD. Lethally irradiated BALB/c mice were transplanted with 5 × 10^6^ TCD-BM from CD45.1^+^ or CD45.2^+^ B6 donors supplemented or not with 0.3 × 10^6^ to 0.5 × 10^6^ total splenocytes. Shortly before transplantation (2–4 hours), mice were injected i.p. with either vehicle (DMSO) or 0.25–0.5 mg/kg CPT every other day for 2–4 weeks. Body weight (**A**) and cGVHD clinical score (**B**) were monitored weekly after allo-BMT. Cumulative scores determined by an independent pathologist were obtained from histological sections and H&E staining that were performed on the indicated tissues in **C** and **D**. Representative photomicrographs (original magnification, ×10) of H&E-stained sections of small intestine and skin from the indicated groups (**E**). Day-40 cumulative flow cytometric analysis of the indicated thymic (**F**) and splenic (**G**) cell populations. Similar experiments were performed except that vehicle and CPT administration was delayed until 28–30 days after BMT, and body weights and cGVHD clinical scores were monitored weekly following BMT (**H**). Data in **A**–**E** represent 2 independent experiments (BM + vehicle, *n* = 3–7; BM + CPT, *n* = 3–6; BM + spleen + vehicle, *n* = 8–15; BM + spleen + CPT, *n* = 9–13). Data in **H** represent 3 independent experiments (BM only, *n* = 5; BM + spleen + vehicle, *n* = 20; BM + spleen + CPT, *n* = 19). Significance was determined using mixed-model tests for clinical scores and body weights and 1-way ANOVA for all other data. *P < 0.05, **P < 0.01, and ***P < 0.001. Rx, treatment; Sp, spleen; Veh, vehicle.

We next investigated whether this strategy would be beneficial for cGVHD prevention or treatment. CPT at low doses was able to effectively prevent cGVHD development in mice, as reflected by clinical manifestations and pathological damage in the GVHD target organs skin and small intestine (Figure 5, A–E). At a late phase of cGVHD, we found that the recipient mice treated with CPT had significantly increased numbers of CD4^+^CD8^+^ thymocytes and significantly reduced CD4^+^ Tfh and CD8^+^ Tfh-like cells (Figure 5, F and G). In addition to cGVHD prevention, delayed CPT administration was able to effectively alleviate the severity of established cGVHD (Figure 5H). To extend our finding, we tested the efficacy of CPT using a classic model of cGVHD, in which cutaneous fibrosis develops after MHC-matched BMT (53). CPT administration starting on the day of BMT substantially alleviated cGVHD severity (Supplemental Figure 10, A–C), consistent with improved thymic CD4^+^CD8^+^ and splenic B cell reconstitution in these recipients (Supplemental Figure 10, D and E). Although CPT treatment increased CD8^+^ T cell production of IFN-γ, it attenuated CD4^+^ T cell pathogenicity, as reflected by more Foxp3-expressing, but fewer IFN-γ–producing, cells in the CD4^+^ population (Supplemental Figure 10F).

**Figure 6:**
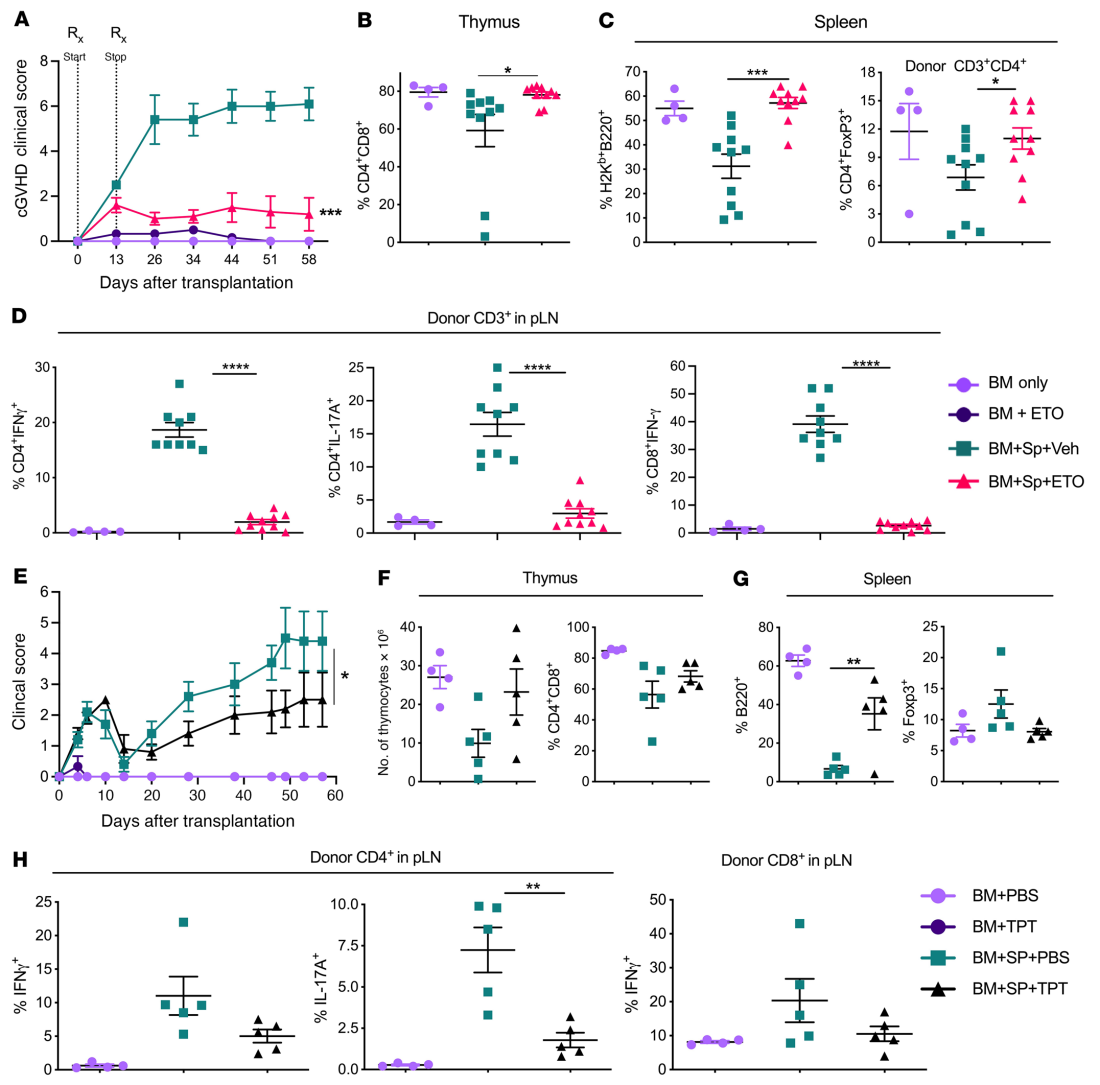
ETO and TPT prevent cGVHD. (**A**–**D**) cGVHD BMT was performed similarly to the procedure described in Figure 4, except mice were given donor BM and splenocytes from WT-B6 mice and supplemented or not with vehicle or 5 mg/kg ETO i.p. starting on day 0 and then every other day until day 14 after BMT. The cGVHD clinical score was monitored weekly (**A**), and the frequencies of thymic CD4^+^CD8^+^ (**B**) and splenic B220^+^ and CD4^+^FoxP3^+^ populations (**C**) were determined. pLN populations of T cells producing cytokines were analyzed on approximately day 60 after BMT (**D**). Data are from 2 independent experiments (BM only, *n* = 4; BM + ETO, *n* = 3; BM + spleen + vehicle, *n* = 10; BM + spleen + ETO, *n* = 10). (**E**–**G**) In a similar BMT setting, the recipient mice were i.p. injected with TPT at 0.3 mg/kg every other day starting on the day of BMT for 10 days. The cGVHD clinical score was monitored weekly (**E**), and the absolute number of thymocytes and the frequency of thymic CD4^+^CD8^+^ (**F**) and splenic B220^+^ and CD4^+^Foxp3^+^ cells (**G**) were analyzed on day 60 after BMT. Cytokine production by donor CD4^+^ and CD8^+^ T cells in pLNs were analyzed on day 60 after BMT (BM only, *n* = 4; BM + TPT, *n* = 3; BM + spleen + PBS, *n* = 5; BM + spleen + TPT, *n* = 5). Significance was determined using mixed-model tests for clinical scores and 1-way ANOVA for all other data. *P < 0.05, **P < 0.01, ***P < 0.001, and ****P < 0.0001.

To determine whether another drug that could also target Fli-1 could reduce cGVHD, we tested an alternative, more clinically relevant drug (ETO) as a potential translational strategy. We found that ETO inhibited Fli-1 expression in both Jurkat cells and polyclonally activated human PBMCs compared with vehicle controls (Supplemental Figure 11), in agreement with a previous study that identified ETO as a Fli-1 inhibitor in murine erythroleukemia cells (15). When used as a prophylactic strategy in a cGVHD mouse model, ETO treatment was able to dramatically reduce cGVHD severity (Figure 6A) and was associated with significant increases in thymic CD4^+^CD8^+^ and splenic B220^+^ and CD4^+^FoxP3^+^ cell populations 60 days after BMT (Figure 6, B and C). We also observed a significant reduction in CD4^+^IFN-γ^+^, CD8^+^IFN-γ^+^, and CD4^+^IL-17A^+^ T cell frequencies in pLNs (Figure 6D). TPT, another drug with previously demonstrated Fli-1–inhibitory activity (16), was also able to reduce Fli-1 expression in human Jurkat cells (Supplemental Figure 12A) and attenuate cGVHD (Figure 6E), including improved thymic and B cell reconstitution, and reduced IL-17A in CD4^+^ T cells (Figure 6, F–H). Further, TPT reduced the frequencies of Tfh-like, IFN-γ^+^, IL-2^+^, and IL-17A^+^ CD4^+^ T cells in the spleen,and reduced the frequencies of donor splenocyte–derived CD4^+^ and CD8^+^ T cells in pLNs (Supplemental Figure 12, B–F). Overall, these results suggest that targeting Fli-1 using low-dose CPT, TPT, or ETO is effective in preventing and/or reversing cGVHD, and this effectiveness can be attributed, at least in part, to Fli-1 inhibition on lymphocytes, since these drugs have established Fli-1–inhibitory activity, as shown in this study and others (15, 16).

### Inhibition of Fli-1 prevents aGVHD and preserves the GVL effect.

**Figure 7:**
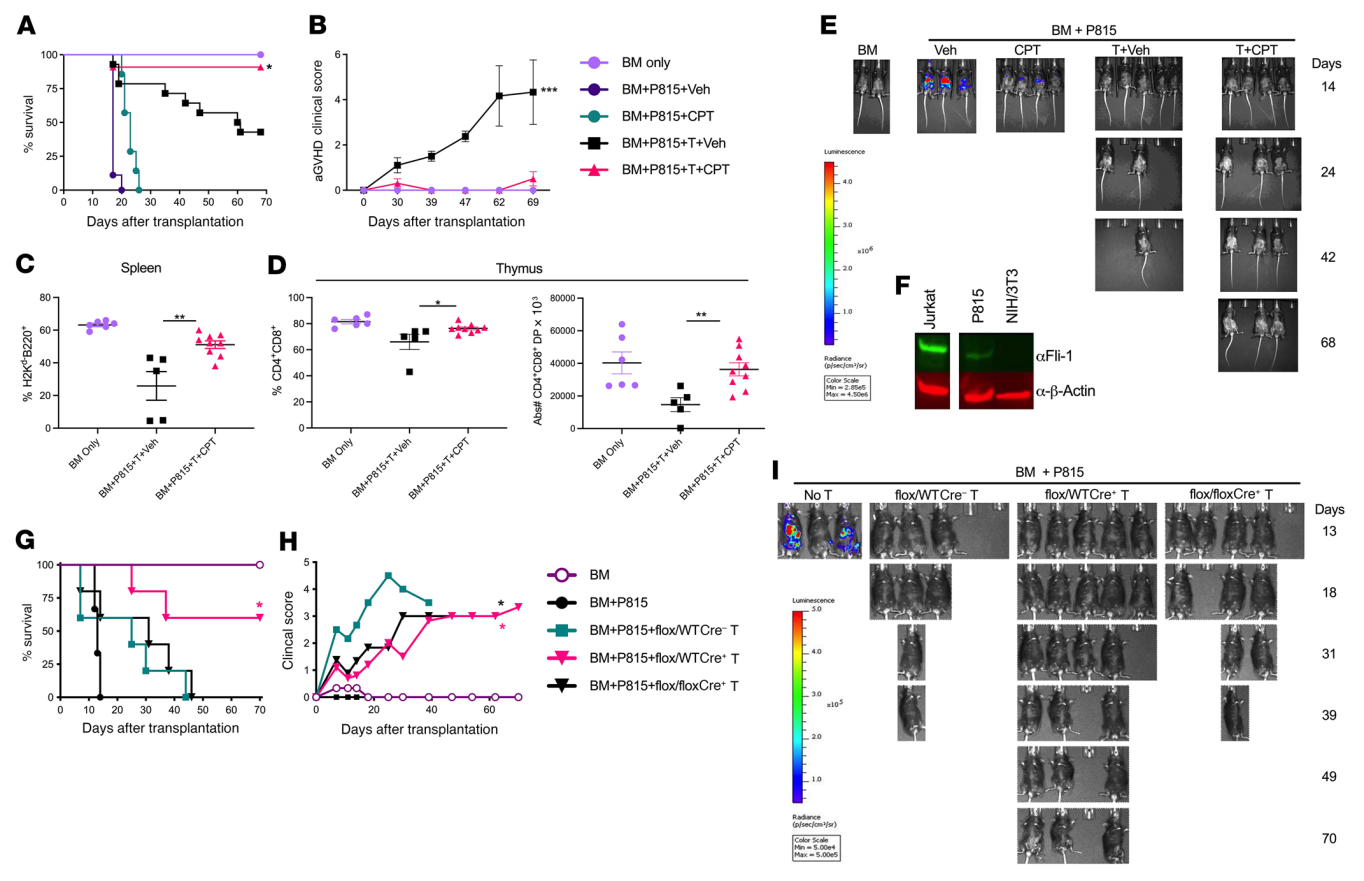
Inhibition of Fli-1 prevents aGVHD and preserves the GVL effect. (**A**–**F**) Lethally irradiated B6D2F1 mice were transplanted with 5 × 10^6^ TCD-BM cells from CD45.1 or CD45.2 B6 donors supplemented or not with 3 × 10^6^ purified total T cells from CD45.2 B6 donors. Three of the 4 groups of mice were also supplemented with 5,000 P815 at the time of BMT and received vehicle, 0.25 mg/kg CPT only, mature T cells plus vehicle (T+Veh), or mature T cells plus 0.25 mg/kg CPT (T+CPT) on day 0 and then every other day until day 28 after BMT. Recipient survival (**A**) and aGVHD clinical scores (**B**) were monitored following BMT. At the experimental endpoint (~day 80) donor splenic H2K^d–^B220^+^ (**C**) and thymic CD4^+^CD8^+^ double-positive (DP) (**D**) cell populations were analyzed by flow cytometry. An IVIS 200 imager was used to periodically monitor firefly-luciferase expression in transplanted P815 cells in recipient mice, which were injected with d-luciferin substrate at each imaging time point (**E**). Western blot analysis of the indicated proteins and tumor cell lines after 24 hours in culture (**F**). α, anti-. Data in **A**–**E** represent 3 independent experiments (BM only, *n* = 6; BM + P815 + vehicle, *n* = 9; BM + P815 + CPT, *n* = 7; BM + P815 + T cells + vehicle, *n* = 14; BM + P815 + T cells + CPT, *n* = 11). Data in **F** are from an individual Western blot. (**G**–**I**) Purified T cells isolated from *Fli1^WT^*, *Fli1^fl/WT^*, or *Fli1^fl/fl^* donor mice plus WT TCD-BM were transferred into lethally irradiated B6D2F1 mice. On the day of BMT, 5,000 luciferase-transduced P815 cells were i.v. injected into these recipients. Recipient survival rates (**G**), clinical scores (**H**), and P815 tumor growth (**I**) were monitored following BMT (BM only, *n* = 3; BM + P815, *n* = 3; BM + P815 + *Fli1^WT^*, *n* = 5; BM + P815 + *Fli1^fl/WT^*, *n* = 5; BM + P815 + *Fli1^fl/fl^*, *n* = 5). Significance was determined using mixed-model tests for clinical scores, a log-rank test for survival data, and a 1-way ANOVA for all other data. *P < 0.05, **P < 0.01, and ***P < 0.001.

To determine the effect of low-dose CPT on the GVL effect, we used a haploidentical murine model of aGVHD supplemented with an aggressive P815 mastocytoma. In this model, mice that received T cell–depleted BM (TCD-BM) without mature T cells succumbed rapidly to P815-mediated mortality, whereas mice that were given mature T cells from allo-BMT were protected against P815 outgrowth (Figure 7A). Here, allo-BMT recipients given TCD-BM, P815, mature T cells, and vehicle developed moderate-to-severe aGVHD leading to significant GVHD-related mortality. In contrast, recipient mice under the same conditions but treated with low-dose CPT had significantly better survival (Figure 7A), reduced aGVHD clinical scores (Figure 7B), and improved thymic CD4^+^CD8^+^ reconstitution and higher donor-derived splenic B220^+^ B cell reconstitution compared with vehicle-treated mice (Figure 7, C and D). Importantly, while mice given TCD-BM plus P815 had rapid P815 outgrowth (100% mortality by day 14), low-dose CPT–treated mice that received mature allogeneic T cells had complete protection against P815 relapse (0 of 13 by day 80), similar to their vehicle-treated counterparts. To examine the direct effects of low-dose CPT against P815 itself, we also treated mice given TCD-BM and P815 with CPT without mature T cells, which resulted in early tumor protection, but ultimately did not directly prevent tumor relapse (100% mortality by day 30) (Figure 7, A and E). P815 cells expressed a low amount of Fli-1 compared with Jurkat cells (Figure 7F). Consistently, recipient mice administered CPT showed an intact GVL response against B cell lymphoma (A20) after allo-BMT, along with significantly reduced GVHD clinical scores (Supplemental Figure 13).

Cyclosporine is a classic immunosuppressive drug for GVHD prophylaxis. Post-transplantation cyclophosphamide (PTCy) or bendamustine has been shown to be effective in controlling GVHD development (54). We thus attempted to compare Fli-1 inhibitors with these “standard” treatments. We observed that short-term treatment with CPT (4 doses) or ETO (2 doses) early after BMT effectively attenuated aGVHD severity (Supplemental Figure 14, A–C). The recipient mice administrated CPT for 2 weeks showed the most favorable outcomes, reflected by the best survival rates, the lowest clinical scores, and no leukemia relapse (Supplemental Figure 14, D–G). Importantly, using the same treatment schedule as for PTCy, 2 doses of CPT or ETO administered on days 3 and 4 were sufficient to prevent GVHD and leukemia relapse. In contrast, some of the recipients treated with bendamustine succumed to GVHD, while some of the mice treated with PTCy or cyclosporine experienced leukemia relapse. Furthermore, long- or short- term administration of CPT, ETO, or TPT did not show toxicity to hematopoietic stem cells (HSCs), as reflected by similar numbers of donor-derived HSCs in the recipients’ BM (Supplemental Figure 15, A–C). Inhibition of Fli-1 with CPT, ETO or TPT did not delay myeloid cell reconstitution, or even improved CD11b^+^ cell reconstitution in PB and spleens from mice treated with CPT or ETO for 2 weeks (Supplemental Figure 15, D and E).

We then tested the ability of T cells with heterozygous or homozygous *Fli1* deficiency to mediate the GVL effect against P815 and found that mice given *Fli1^fl/WT^*Cre^+^ T cells were able to survive long term (Figure 7G), while also maintaining a lower aGVHD clinical score (Figure 7H) and a strong GVL effect against P815 (Figure 7I) compared with mice that received WT T cells. Consistently, the recipients of *Fli1^fl/fl^*Cre^+^ T cells also showed improved clinical manifestations and maintained GVL responses. A recent study demonstrated that Fli-1 represses effector CD8^+^ T cells responses during anti-infection and antitumor responses (14). We further studied the role of Fli-1 in regulating CD8^+^ T cell responses during GVL activity after allo-BMT. Compared with WT control CD8^+^ T cells, *Fli1*-deficient CD8^+^ T cells in both the spleen and liver had an enhanced ability to become memory precursor effector cells (KLRG1^–^CD127^+^) and expressed lower Lag3 in the liver. With the exception of IFN-γ, both control CD8^+^ T cells and *Fli1*-deficient CD8^+^ T cells had comparable expression levels of granzyme B, TNF-α, CD107a, Fas-L, PD-1, and CXCR3 (Supplemental Figure 16). Taken together, these results indicate that T cells with either heterozygous or homozygous *Fli1* deficiency maintained their GVL activity. Additionally, we found that targeting Fli-1 using low-dose CPT or ETO was an effective strategy to reduce aGVHD severity and lethality after allo-BMT, while preserving the ability of alloreactive T cells to prevent leukemia relapse.

### CPT inhibits Fli-1 on human T cells and reduces GVHD in a xenograft model.

**Figure 8:**
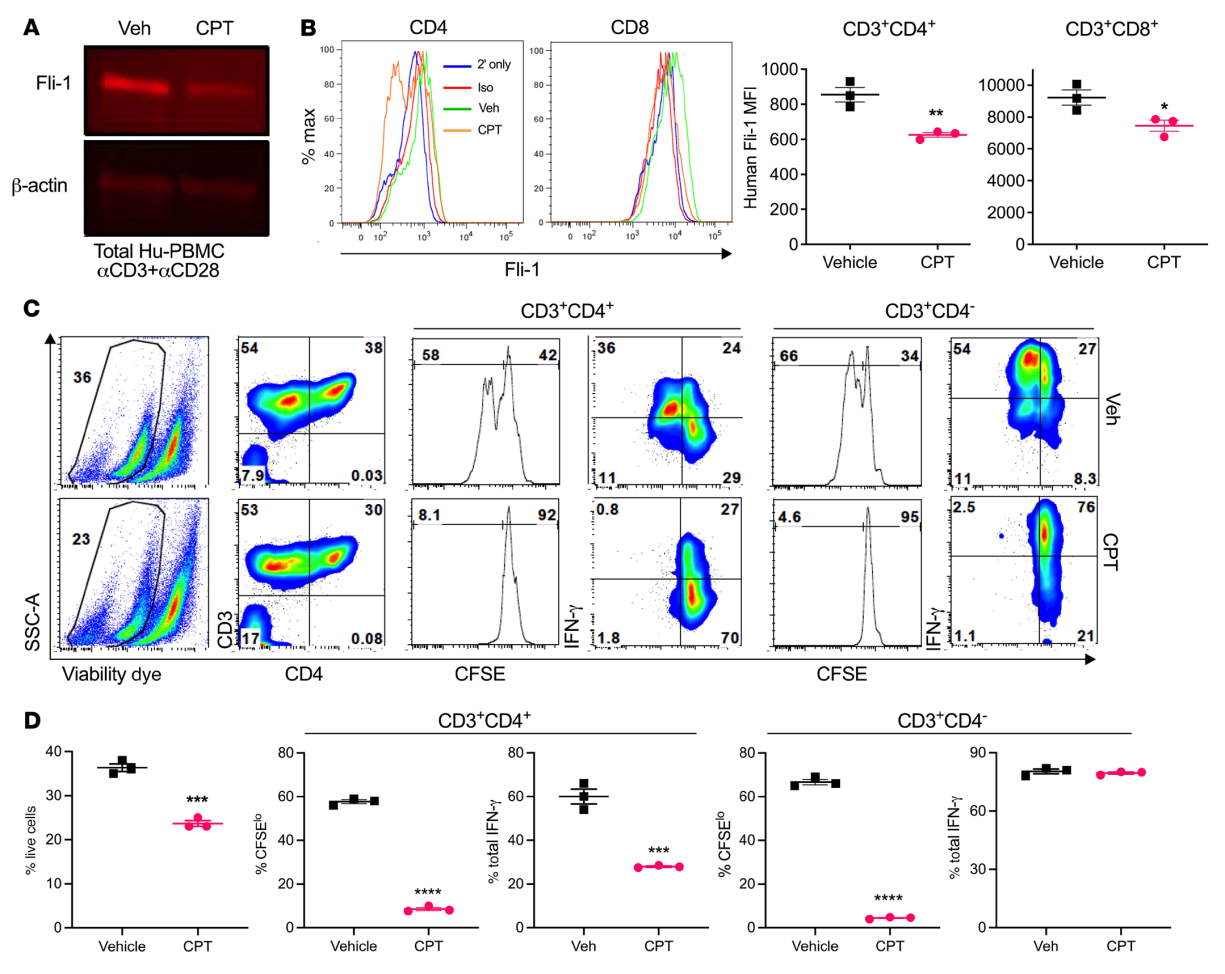
CPT acts as a Fli-1 inhibitor on human T cells and reduces their proliferation in vitro. Total human PBMCs isolated from healthy donors were CFSE labeled and activated in vitro via soluble anti-CD3/anti-CD28 (2 μg/mL) and cocultured with DMSO (vehicle) or 15 nM CPT for 3 days. On day 3, cultures were harvested and lysed for Western blot analysis of Fli-1 protein expression, with β-actin as the loading control (**A**). Representative flow cytometric histograms show intracellular Fli-1 expression in CD3^+^CD4^+^ and CD3^+^CD4^–^CD8^+^ gated T cells treated with vehicle or 15 nM CPT (left) and representative Fli-1 MFI values (right); isotype (Iso) control (red line); secondary antibody only (blue line); vehicle-treated cells (green line); CPT-treated cells (orange line) (**B**). Max, maximum. Representative flow cytometric plots show proliferation (CFSE dilution) and IFN-γ cytokine production in human T cells (**C**). SSC-A, side scatter area. Representative frequencies of live cells in culture (left), CD4 proliferation and cytokine production (middle), and CD8 proliferation and cytokine production (right) are shown (**D**). Data represent 2 independent experiments, each performed in triplicate except for Western blotting, in which triplicate wells were combined into single lysates for each condition, and 2 independent blots were performed. Significance was determined using an unpaired, 2-tailed Student’s *t* test. *P < 0.05, **P < 0.01, ***P < 0.001, and ****P < 0.0001.

To further increase the clinical relevance of our study, we tested CPT in human cells and found that CPT at a very low dose was able to potently inhibit Fli-1 protein levels in a human transformed T cell line (Jurkat) (Supplemental Figure 17A). Jurkat cells had reduced growth and markedly induction of apoptosis via CPT by culture day 3 compared with vehicle treatment (Supplemental Figure 17, B and C). CPT was also a potent inhibitor of Fli-1 in polyclonally stimulated human PBMCs in vitro (Figure 8A). To further confirm Fli-1 protein expression specifically in human T cells, we used an available human anti–Fli-1 flow cytometry antibody. In agreement with the Western blot data, we found significantly reduced expression of Fli-1 in both CD3^+^CD4^+^ and CD3^+^CD4^–^ T cells that had been treated with CPT compared with cells treated with vehicle (Figure 8B). Low-dose CPT treatment of activated human PBMCs was able to significantly reduce T cell survival and proliferation, but was able to highly preserve T cell IFN-γ production, especially in CD8^+^ T cells (Figure 8, C and D).

**Figure 9:**
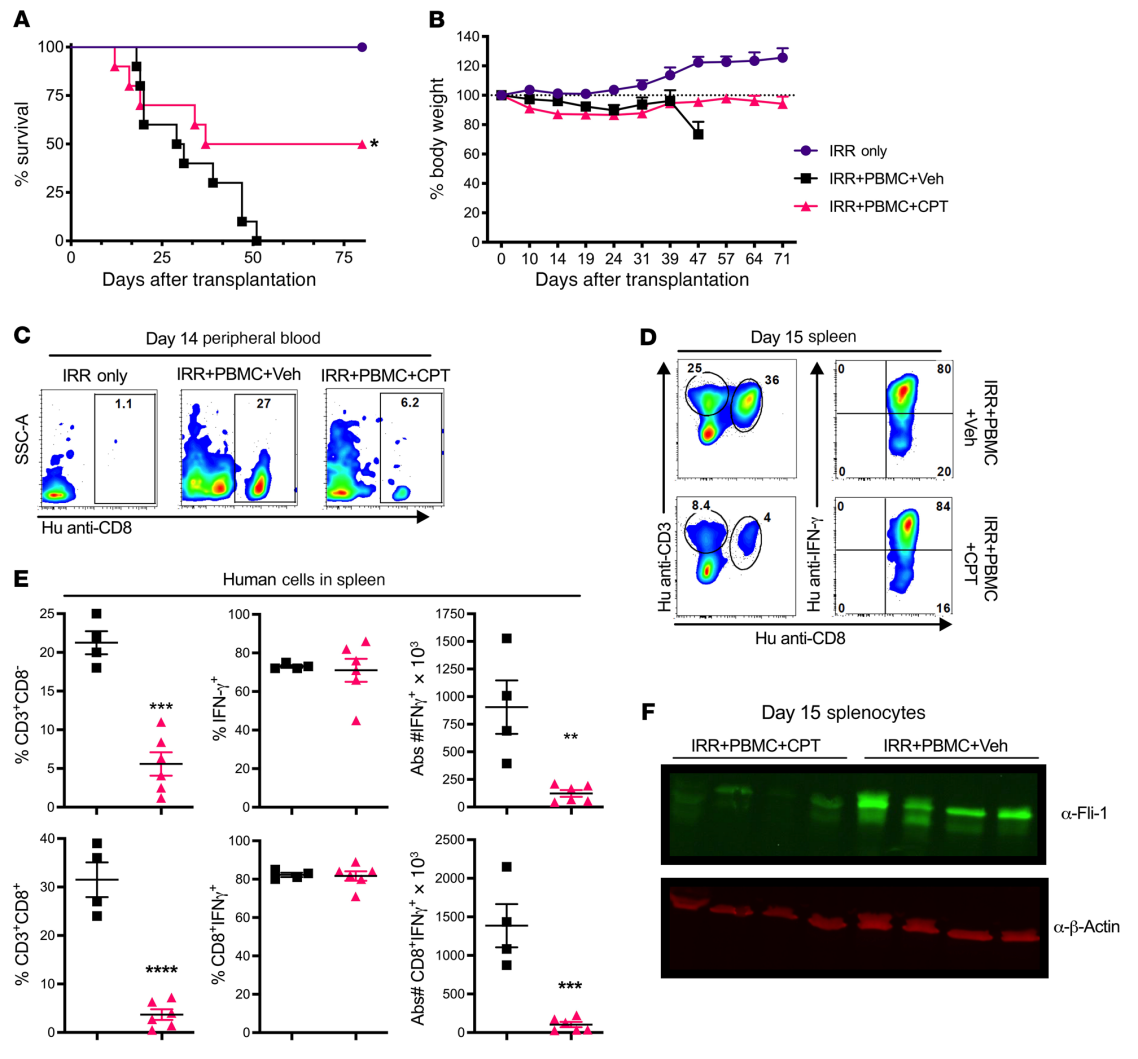
CPT inhibits human Fli-1 and reduces GVHD in a xenograft model. HLA-A2^+^ NSG mice were sublethally irradiated (250 cGy) and transplanted with 8 × 10^6^ to 10 × 10^6^ total human PBMCs from a healthy donor (HLA-A2^–^) to induce human GVHD. These mice received vehicle or CPT at 0.25–0.5 mg/kg on day 0, which was then every other day until day 14 after BMT. Recipient survival rates (**A**) and body weights (**B**) were monitored up to 80 days after transplantation. Peripheral blood staining of human (Hu) CD8^+^ T cells on day 14 after transplantation (**C**). Staining of human T cells within spleens of xenograph recipients on day 15 after transplantation (**D**), and percentage and number of human IFN-γ–producing CD3^+^CD8^–^ T cells (top) and human IFN-γ–producing CD3^+^CD8^+^ T cells (bottom) (**E**). Western blot of day-15 splenic whole-cell lysates from 4 vehicle-treated and 4 CPT-treated xenografted mice using the indicated primary antibodies (**F**). Data in **A** and **B** represent 2 independent experiments (IRR only, *n* = 6; IRR + PBMCs + vehicle, *n* = 10; IRR + PBMCs + CPT, *n* = 10). Data in **C**–**F** were collected from 1 set of mice belonging to 2 independent experiments. Significance was determined using mixed-model tests for body weight, a log-rank test for survival data, and an unpaired, 2-tailed Student’s *t* test for all other data. *P < 0.05, **P < 0.01, ***P < 0.001, and ****P < 0.0001. IRR, irradiation.

We then tested whether low-dose CPT would be able to reduce GVHD in a human-to-mouse xenograft model. Here, low-dose CPT administration given prophylactically to mice for 2 weeks led to significantly increased survival rates and body weights compared with vehicle-treated mice (Figure 9, A and B). PB taken from recipient mice on day 14 after transplant confirmed human T cell engraftment (Figure 9C). Significant reductions in the frequency and number of both CD3^+^CD8^–^ and CD3^+^CD8^+^ populations were observed, although their ability to produce IFN-γ^+^ was not reduced on a per-cell basis (Figure 9, D and E). We then confirmed that CPT also acts as a Fli-1 inhibitor in vivo, as cells extracted from splenocytes of mice treated with CPT showed an obvious reduction in Fli-1 protein levels (Figure 9F). To study how CPT affects human Tregs, we performed a separate xenograft GVHD experiment and observed improved body weight maintenance and survival of mice that received CPT treatment (Supplemental Figure 18A), but also found that the frequencies of human Tregs in CPT-treated mice were not reduced in the spleens of recipient mice and were modestly elevated in recipients’ livers compared with vehicle-treated mice (Supplemental Figure 18, B and C). In addition, CD25^+^CD4^+^ and CD25^+^CD8^+^ T cells expressed the highest levels of Fli-1, especially after activation, while Foxp3^+^ and CD25^–^Foxp3^–^ cells maintained the lowest Fli-1 expression, which might be explained by the inhibition of Fli-1 that spared Tregs (Supplemental Figure 18D). Consistently, we found no evident impairment of body weight maintenance or myeloid cell reconstitution after a full course of CPT treatment (Supplemental Figure 18, E–G). Cumulatively, these data indicate an ability of CPT to act as a Fli-1 inhibitor on primary human lymphocytes and that CPT can reduce human T cell proliferation as well as improve the survival of graft recipients in a xenograft model.

## Discussion

The specific role of Fli-1 in primary CD4^+^ T cells has not been studied in depth to date, especially not in allo-HCT conditions. Our findings, combined with those in the previous literature, allow us to posit several potential mechanisms that would explain how Fli-1 regulates the allogeneic T cell response during GVHD development.

Particularly interesting in our study was the finding that, in genotype-matched spleen and BM cGVHD transplants, T cell–specific heterozygous Fli-1 reduction led to distinctly different outcomes with regard to clinical score and T cell phenotypes when compared with both groups: mice with homozygous reduction of Fli-1 activity and WT mice. However, these disparate cGVHD clinical score outcomes between the heterozygous and homozygous groups were largely diminished when the source of donor BM was changed from *Fli1^fl/fl^*Cre^+^ to WT marrow, although several differences still remained regarding T cell phenotypes. When we switched *Fli1^fl/fl^*Cre^+^ BM to WT BM, we noted a substantial increase in the frequency of CD4^+^CD8^+^ thymocytes during cGVHD. Indeed, a previous group discovered that germline heterozygous mutation of *Fli1* resulted in normal thymus development, but homozygous mutation resulted in a significant reduction in thymocyte numbers that was attributed to defects in prethymic T cell progenitors (26). This report is consistent with our findings that a homozygous, but not a heterozygous, reduction of Fli-1 activity on donor BM–derived T cells could impair the frequency of CD4^+^CD8^+^ thymic T cell repopulation after allo-BMT, suggesting that at least 1 allele of *Fli1* was required for optimal thymic reconstitution. Bulk RNA-Seq and qRT-PCR analyses suggested that, upon T cell activation with alloantigen, Fli-1 can contribute to the regulation of genes associated with activation and inflammation, as well as anti-inflammatory T cell genes that can contribute to and suppress GVHD development, respectively.

The scRNA-Seq analysis of the donor T cells isolated from the allo-BMT recipients indicated a major difference in the transcriptional regulation by Fli-1 in CD4^+^ versus CD8^+^ T cells and in the *Fli1* gene dose-dependent modification of transcriptional pathways in *Fli1^Het^* versus *Fli1^KO^* T cells. Fli-1 was deemed to play a distinct role in regulating gene transcription in CD4^+^ versus CD8^+^ T cells, in that more activation and fewer exhaustion gene pathways were enriched in CD8^+^ *Fli1*-deficient T cells, whereas fewer Th1/Th17 pathogenic pathways were enriched in CD4^+^ *Fli1-*deficient T cells. Overall, *Fli1* deficiency increased gene enrichment in the OXPHOS pathway in both CD4^+^ and CD8^+^ T cells and substantially reduced gene enrichment in the glycolysis pathway in CD4^+^ T cells. We and others reported that alloreactive T cells upregulate essential metabolic pathways, in which glycolysis manifests as a major source of energy for GVHD-inducing T cells (55). On the other hand, lower levels of glycolysis and increased levels of OXPHOS are beneficial to the generation of long-lived memory T cells for persistent antitumor activity (56). Thus, metabolic modification and differential regulation of CD4^+^ versus CD8^+^ T cell responses in *Fli1* deficiency may be beneficial for maintaining GVL activity, while attenuating GVHD pathogenicity. Consistent with the lowest pathogenicity of *Fli1^Het^* T cells for GVHD induction, we observed that *Fli1^Het^* CD4^+^ T cells had the lowest Th1/Th17 pathway enrichment and TCR pathway downstream gene expression. Further study is required to define the mechanism by which different doses of the *Fli1* gene regulate the expression and function of these TCR downstream TFs in modifying CD4^+^ and CD8^+^ T cell responses.

In a recent report, Chen et al. found that Fli-1 antagonized the differentiation of KLRG1^hi^ Teff cells during acutely resolved infection and also chronic infection mediated by antigen-specific CD8^+^ T cells (14). They elegantly demonstrated that Fli-1 inhibited T effector–like (Teff-like) cell differentiation by coordinating with Runx1 and antagonizing Runx3 function. Interestingly, in our allo-BMT models, *Fli1^KO^* CD8^+^ T cells showed comparable frequencies of KLRG1^+^CD127^–^ Teff cells, but greater frequencies of KLRG1^–^CD127^+^ memory precursor cells, than did WT controls in recipient spleens and livers. *Fli1^KO^* CD8^+^ T cells produced less IFN-γ in recipient spleens and lower levels of Lag3 in recipient livers. Similarly, in our scRNA-Seq analysis, *Fli1^KO^* CD8^+^ T cells showed higher enrichment for memory genes, but lower enrichment for effector pathway genes. On the other hand, *Fli1^Het^* CD8^+^ T cells had more effector but less memory gene enrichment compared with WT controls, suggesting a possible gene dose effect of Fli-1 on CD8^+^ T cell differentiation. Consistent with this study, we found that *Fli1^KO^* CD8^+^ T cells expressed higher levels of Runx3, a TF critical for epigenetic modification and differentiation of CD8^+^ CTLs into effector memory cells and tissue-resident memory cells (51, 52). Furthermore, both *Fli1^Het^* and *Fli1^KO^* CD8^+^ T cells had higher activation but lower exhaustion gene module scores than did WT controls, suggesting that Fli-1 may negatively regulate CD8^+^ T cell function during the allogeneic response. However, since CD4^+^ T cells were the predominant T cell subset that drove GVHD pathogenesis in these tested models, we interpret this to mean that the GVHD alleviation resulted from the reduced CD4^+^ T cell activation in allo-BMT. Under this condition, CD8^+^ T cells did not exhibit higher effector function and maintained greater memory programming in the absence of *Fli1*.

There has still been relatively little progress in the field in the development of a Fli-1–specific inhibitor, despite its known involvement in multiple types of malignancies. Therefore, some of the only pharmacological strategies available to date that can inhibit Fli-1 are known chemotherapy drugs such as CPT, TPT, and ETO. Thus, we used each of these drugs in our study to determine whether targeting Fli-1 pharmacologically would be beneficial in allo-HCT. We observed that CPT did not obviously impair the enzymatic activity of topoisomerase I at low concentrations in activated murine T cells. It was also previously reported that CPT is significantly less effective at preventing the growth of malignant cell lines designed to overexpress Fli-1 (15). The current study further supports our recent report that low doses of CPT or TPT inhibit Fli-1 and significantly attenuate lupus nephritis without liver toxicity or myelosuppression (57). Taken together, these results suggest that CPT or TPT acts through Fli-1 inhibition as an important and currently underappreciated mechanism of action. Nonetheless, beyond targeting Fli-1, we cannot exclude other potential mechanisms by which the topoisomerase inhibitors alleviated GVHD in vivo, given that topoisomerases are involved in DNA repair, replication, and transcription during mitosis (58). Inhibition of topoisomerases by CPT, ETO, or TPT could induce apoptosis of activated T cells during the G_1_ to S-phase transition (59), reduce the expression of MHC-II and costimulatory molecules on APCs (60, 61), or activate the stimulator of IFN genes (STING) pathway (62) and other Fli-1–regulated inflammatory factors, such as CXCR3, IL-6, C16-ceramide, GM-CSF, and miR-17-92 (4–8). All of these pathways have been shown by us and others to critically contribute to GVHD pathogenesis (9, 10–13, 63).

We used low-dose CPT and examined its effect on GVHD prevention and leukemia control. We found in subsequent studies involving allo-HCT experiments that P815 expressed low levels of Fli-1 compared with other cell lines such as the Jurkat cell line. This could potentially explain why there was an early benefit of CPT administration against P815. We observed that the GVL response against P815 or A20 was not impaired by CPT treatment. In agreement with our data showing the ability of CPT to inhibit Fli-1 and preserve the GVL effect, both *Fli1^fl/WT^*Cre^+^ T cells and *Fli1^fl/fl^*Cre^+^ T cells also had the ability to preserve the GVL effect.

In addition, ETO, as well as TPT, was able to reduce Fli-1 expression and cGVHD development through suppression of inflammatory T cell responses, while sparing Tregs in lymphoid tissues, in agreement with a previous report showing that ETO was able to selectively target activated T cells (64). While human effector T cells were reduced in both the spleen and the liver, human CD4^+^FoxP3^+^ Treg were not reduced after CPT treatment, suggesting that CPT can selectively target effector T cells without impairing Tregs. These findings are consistent with our in vitro data showing that inhibition of Fli-1 enhanced murine iTreg numbers and functional molecules. These effects of CPT, TPT, and ETO may be due, at least in part, to a reduction of Fli-1 activity (15, 16). Interestingly, ETO used in the clinic as a myeloablative conditioning regimen has compared favorably with other agents such as cyclophosphamide for the ability to reduce leukemia relapse and GVHD severity (65, 66). Thus, it is worth exploring in future studies whether currently utilized chemotherapeutic agents such as TPT, ETO, and other chemically related drugs (e.g., irinotecan) could be repurposed as strategies to reduce Fli-1 activity and prevent or treat GVHD in the clinical setting. Furthermore, more highly specific Fli-1 inhibitors have recently been identified that will promote the targeting of Fli-1 as an interventional strategy in clinical applications (67).

In preclinical studies, PTCy was found to be effective in preventing GVHD that was largely attributed to selective elimination of alloreactive T cells, functional impairment of alloreactive T cells, and preferential recovery of CD4^+^ Tregs (68, 69). High-dose cyclophosphamide (50 mg/kg) given on day 3 or days 3 and 4 after transplantation was associated with a low incidence of aGVHD but extensive cGVHD in patients following nonmyeloablative HLA-haploidentical HCT. However, malignant relapse was a major reason for treatment failure in these patients with high-risk hematologic malignancies and was possibly caused by cyclophosphamide-mediated deletion of tumor-specific CD8^+^ T cells (54, 70). In the setting of myeloablative conditioning regimens, although relapse rates were reduced, increased GVHD and nonrelapse mortality were observed (71, 72). An alternative strategy to separate T cell GVH and GVL responses is highly warranted. We directly compared the outcomes of CPT, ETO, and TPT versus PTCy treatment using a haploidentical model of GVHD and found that 2 weeks of CPT treatment showed the best outcomes, free of GVHD and leukemia relapse (Supplemental Figure 14, D–G). Two doses of CPT or ETO early after BMT were as effective as PTCy in preventing GVHD and had a less negative impact on the GVL effect and were thus more effective in controlling leukemia relapse. Therefore, targeting Fli-1 with CPT, ETO, or TPT may represent an effective therapeutic approach in GVHD prophylaxis, while maintaining the GVL effect.

In conclusion, we show evidence that Fli-1 plays a critical role in the alloreactive and antigen-specific CD4^+^ T cell response, and based on these results, we show that Fli-1 is a pathogenic factor that can promote inflammatory T cell phenotypes and suppress Tregs, both in vitro and in vivo. Thus, targeting Fli-1 using a pharmacological strategy could be potentially beneficial in the allo-HCT setting by (a) targeting leukemias and lymphomas that overexpress or rely on Fli-1; and (b) targeting pathogenic alloreactive T cells that utilize Fli-1 to some extent for differentiation, survival, or cellular functions. Overall, these results suggest that strategies to reduce Fli-1 expression or transcriptional activity may be a promising area of future research for therapies that aim to reduce GVHD development without compromising the ability of T cells to mediate antileukemia activity. The identification and implementation of specific Fli-1 inhibitors will further promote the translation of our findings into clinical applications.

## Methods

### Experimental mice.

Female and male BALB/c (H-2^d^), C57BL/6 (B6).Ly5.1 (H-2^b^, CD45.1), B6.Ly5.2 (H-2^b^, CD45.2), and (BALB/c x DBA2)F1 (B6D2F1, H-2^b/d^) mice were purchased from Charles River Laboratories. *Rag1^–/–^* (H-2^b^) and NSG mice (NOD.Cg-*Prkdc^SCID^* Il2rg*^tm1Wjl^* Tg(HLA-A/H2-D/B2M)1Dvs./SzJ; stock no. 014570) were purchased from The Jackson Laboratory. *Fli1^fl/fl^* mice on a B6 background were a gift from Xian Zhang’s group (MUSC, Charleston, South Carolina, USA) (19). T cell conditional deletion of *Fli1* exons 3 and 4 was mediated by a Cre/*lox* system utilizing the *CD4* promoter. Homozygous *Fli1* exon 3 and 4 deletion (referred to as *Fli1^fl/fl^* Cre^+^) was mediated via *Fli1^fl/fl^* *CD4*Cre^+^; heterozygous *Fli1* exon 3 and 4 deletion (referred to as *Fli1^fl/WT^* Cre^+^) was mediated via *Fli1^fl/WT^* *CD4*Cre*^+^*; and WT controls (referred to as *Fli1^WT/WT^*) were *Fli1^fl/fl^* CD4Cre^–^, Fli1^fl/WT^ CD4Cre^–^, or Fli1^WT/WT^ CD4Cre^+^. *Fli1^fl/fl^ CD4*Cre^+^ mice were also crossed with Marilyn transgenic mice, described previously (27), to generate HY-antigen–specific T cells with reduced Fli-1 activity. All mice were maintained in a specific pathogen–free facility at an American Association for Laboratory Animal Care–accredited Animal Resource Center at the MUSC and the MCW. Mice were randomly assigned to groups for all relevant experiments, and both female and male donor and recipient mice were tested in genetic and pharmacological experiments.

### Experimental procedures and statistics.

Allo-BMT, the GVL model, treatment with Fli-1–inhibiting drugs, and statistical analyses are described in detail in the Supplemental Methods. RNA-Seq raw data files can be found in the Sequence Read Archive (SRA) database (SAMN30526153, SAMN30526154, SAMN30526155, SAMN30526295, SAMN30526296, and SAMN30526297).

### Study approval.

All animal experiments were approved by the IACUC of the MUSC and the Animal Use Application (AUA) of the MCW.

## Author contributions

SDS participated in experimental design, performed research, collected, analyzed, and interpreted data, performed statistical analysis, and drafted and revised the manuscript. YW participated in experimental design, performed research, collected, analyzed, and interpreted data, performed scRNA-Seq data analysis and statistical analysis, and revised the manuscript. AK analyzed scRNA-Seq data. DB, HJC, MHS, CM, BMM, and HN performed research and interpreted data. CL and KH performed histological scoring of mouse tissues. YBD participated in experimental design, interpreted data, and revised the manuscript. WC interpreted data and revised the manuscript. XZ participated in experimental design, interpreted data, revised the manuscript, and generated genetically modified Fli-1 mice. XZY designed research, interpreted data, performed statistical analysis, and revised the manuscript.

## Supplementary Material

Supplemental data

## Version 1 09/08/2022

In-Press Preview

## Version 2 11/01/2022

Electronic publication
