# Supplementary material for: The druggable transcription factor Fli-1 regulates T cell immunity and tolerance in graft-versus-host disease
Source: J Clin Invest. 2022 Nov 1;132(21):e143950. doi: 10.1172/JCI143950 (PMC9621143; doi:10.1172/JCI143950)
Supplement: Supplemental data [file jci-132-143950-s022.pdf]

Supplementary Materials

Figure S1. Characteristics of *Fli1* conditional knock-out (KO) mice.

Figure S2. Fli-1 on splenocytes-derived T cells contributes to cGVHD.

Figure S3. Fli-1 inhibits antigen-specific iTreg generation while promoting IL-2 secretion and Th17 differentiation in vitro.

Figure S4. Fli-1 regulates T-cell pathogenicity in murine colitis.

Figure S5. Fli-1 contributes to regulation of genes involved in Treg and effector T-cell development and function.

Figure S6. Predicted differential upstream regulator pathways between *Fli1 flox/wt* Cre+ and *flox/flox* Cre+ T cells during GVHD.

Figure S7. Fli-1 regulates gene transcription involved in activation, differentiation and function of CD8 T cells.

Figure S8. Low-dose camptothecin (CPT) inhibits Fli-1, reduces tumor growth, and murine T-cell proliferation.

Figure S9. Low-dose CPT inhibits activation of T cells and suppresses effector T cells while sparing Tregs in vitro.

Figure S10. Low-dose CPT effectively prevent cGVHD development after MHC-matched BMT.

Figure S11. Etoposide inhibits Fli-1 in activated human PBMC and Jurkat cells.

Figure S12. Topotecan inhibits T-cell allogeneic response.

Figure S13. Camptothecin (CPT) treatment preserves T-cell mediated GVL activity.

Figure S14. Short-term inhibition Fli-1 with Camptothecin (CPT) or Etoposide (ETO) effectively controls GVHD whiles sparing GVL activity.

Figure S15. Inhibition of Fli-1 with Camptothecin (CPT), Etoposide (ETO) or Topotecan (TPT) did not adversely impact hematopoietic stem cells (HSCs) and myeloid cells.

Figure S16. Fli-1 expression in donor T cells is not essential for GVL response and CD8 T-cell function.

Figure S17. Camptothecin inhibits Fli-1 expression and growth in human Jurkat cells.

Figure S18. CPT treatment had little impacts on Treg and myeloid cell populations.

Table S1. Primers used in the study.

Figure S1

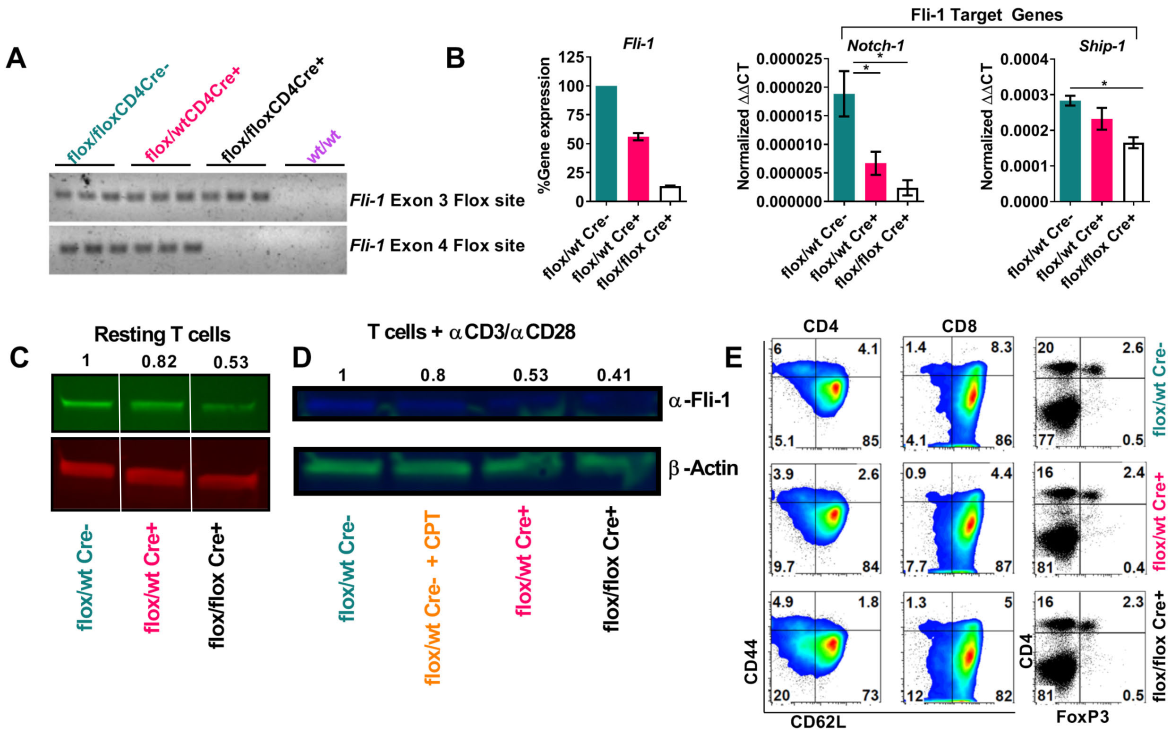

Figure S1. Characteristics of fli-1 conditional knock-out (KO) mice. PCR probes specific to the consensus LoxP sequence flanked by primers specific to either Fli-1 exon 3 flox site or exon 4 flox site were used to determine the efficiency of Cre-recombinase mediated recombination of Fli-1 exons. Genomic DNA was isolated from purified naïve $CD4^{+}$ T cells of $Fli1^{wt/wt}$, $Fli1^{flox/wt}$, or $Fli1^{flox/flox}$ mice and amplified using LoxP probes specific for exon 3 and exon 4 (n=2-3 per condition) **(A)**. qRT-PCR primers designed to detect Fli-1, Notch-1, or Ship-1 gene expression were used to amplify cDNA converted from total mRNA of *in vitro* polyclonally stimulated T cells from the indicated genotypes (n=2-3 per condition) **(B)**. Western blot analysis of Fli-1 and β-actin loading control performed on lysates from resting purified T cells **(C)** and polyclonally activated purified T cells after 48h stimulation **(D)**. A thin white line in figure C indicates separated lanes that were run on the same gel but noncontiguous. Some activated T-cell cultures were incubated with 15nM CPT for 48h to assess impact of CPT on Fli-1 protein level of activated T cells (lane 2). Splenocytes from the indicated donor genotypes were stained with the indicated surface or intracellular markers to determine potential differences in the baseline T-cell phenotype. Across >6 donor mice, no significant differences were observed in naïve/memory/central memory, or nTreg populations **(E)**. Data in A, C, D represent an individual experiment, data in B represent two independent experiments. Data were shown as mean ± SEM by one-way ANOVA and Tukey's HSD post hoc analysis. *$p<0.05$.

Figure S2

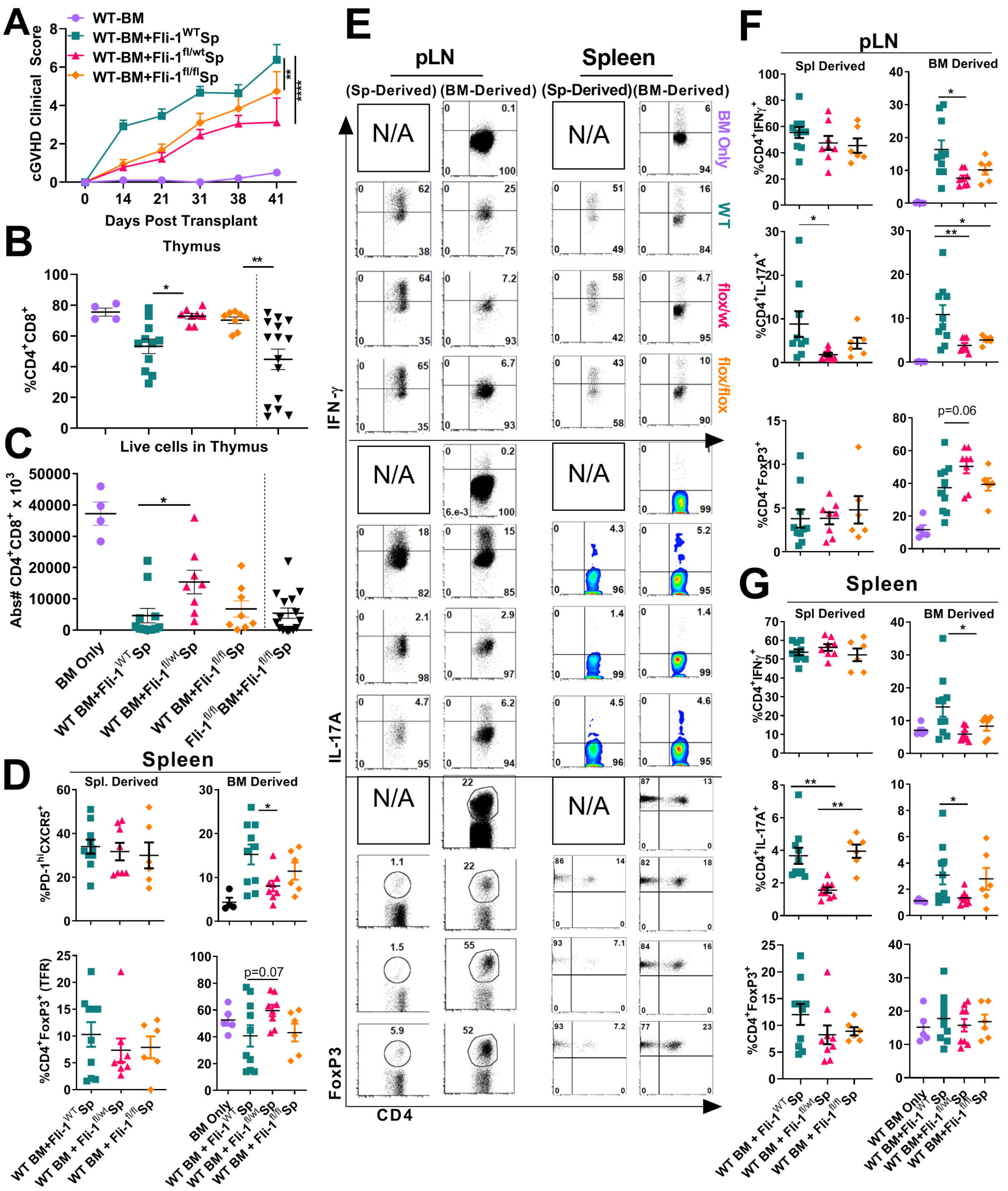

Figure S2. Fli-1 on splenocytes-derived T cells contributes to cGVHD. Similar experiments were performed as in Fig. 2, except that donor BM source for all BALB/c recipients was changed to WT CD45.1$^+$ B6 donors, supplemented with or without 0.5 x 10^6 donor splenocytes from *Fli1*$^{wt/wt}$, *Fli1*$^{flox/wt}$, or *Fli1*$^{flox/flox}$ donors. cGVHD clinical score was monitored following BMT **(A)**. Cumulative thymic CD4$^+$CD8$^+$ populations were analyzed ~day 40 post transplant via flow cytometry **(B-C)**. The data in *Fli1*$^{flox/flox}$BM+*Fli1*$^{flox/flox}$ Sp in B-C was derived from experiments in figure 2 to compare to the mice that received *Fli1*^{WT} BM and *Fli1*$^{flox/flox}$Sp (WT BM+*Fli1*$^{flox/flox}$Sp). Splenocytes of recipients were analyzed for the indicated T_{FH}-like and T_{FR} surface and intracellular markers and cumulative frequencies are shown **(D)**. Representative flow cytometry plots show splenocyte derived (CD45.1-) or BM-derived (CD45.1+)

donor T-cell populations within peripheral lymph nodes (pLN) or spleen **(E)**. The first four plots (IFNγ+) are labeled with the corresponding genotype, and the order is the same in subsequent sets of 4 plots. pLN **(F)** and spleens **(G)** of recipients were analyzed for the indicated intracellular cytokines and cumulative frequencies are shown. Data represent two independent experiments (WT-BM-Only n=4; WT-BM+*Fli1*^{WT}-Sp n=11; WT-BM+ *Fli1*$^{\text{flox/wt}}$-Sp n=8; WT-BM+ *Fli1*$^{\text{flox/flox}}$-Sp n=8). Data were shown as mean ± SEM by one-way ANOVA and Tukey's HSD post hoc analysis. *p<0.05, **p<0.01, ***p<0.001.

Figure S3

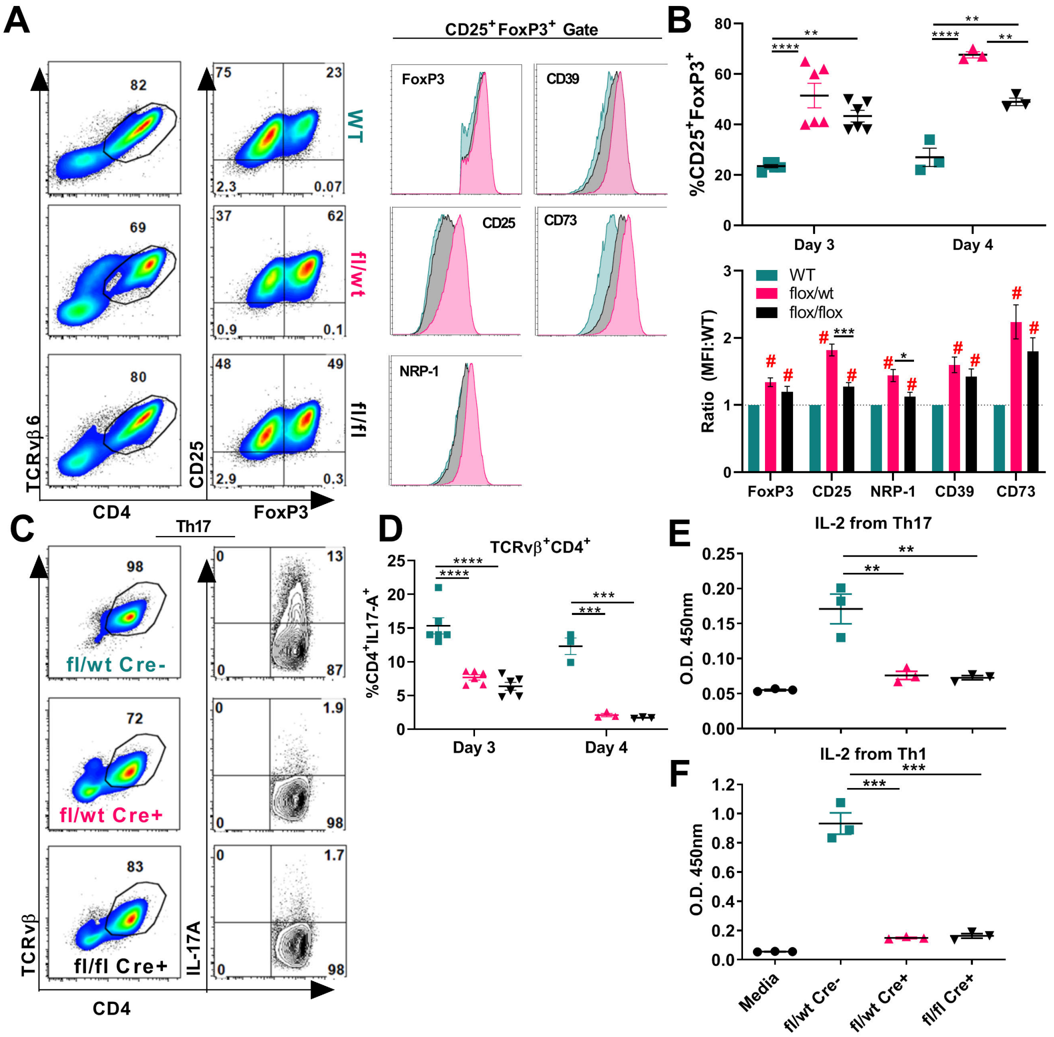

Figure S3. Fli-1 inhibits antigen-specific iTreg generation while promoting IL-2 secretion and Th17 differentiation in vitro. CD8 and CD25-depleted splenocytes from *Fli1*$^{wt/wt}$, *Fli1*$^{flox/wt}$, and *Fli1*$^{flox/flox}$ TCR-tg mice were polarized into iTreg using 0.5μg/ml HY-peptide, 5ng/ml TGF-β, and 2ng/ml IL-2. Cultures were analyzed on days 3 and 4 for frequency of iTreg (TCRvβ6+CD4+CD25+FoxP3+) **(A)** and surface Treg functional molecules CD39, CD73, and NRP-1 **(B)**. Cumulative *Fli1*$^{wt/wt}$ MFI values for CD25, FoxP3, NRP-1, CD39, and CD73 were set to "1" as a baseline comparison, and the ratio of *Fli1*$^{flox/wt}$ and *Fli1*$^{flox/flox}$ MFI values over *Fli1*$^{wt/wt}$ values was calculated and graphed. Hashtag symbol indicates statistical significance (p<0.05) between *Fli1* WT and indicated groups was reached within each individual experiment. The same TCRtg splenocytes were polarized into Th17 via addition of 0.5ug/ml HY-peptide, 10μg/ml α-IFN-γ, 10ng/ml IL-6, and 5ng/ml TGF-β and analyzed on days 3 and 4 for frequency of Th17 cells (TCRvβ+CD4IL-17A+) **(C)** and cumulative results are graphed in **(D)**. Prior to re-stimulation, culture supernatants from each condition were collected and assayed for IL-2 cytokine abundance using ELISA **(E-F)**. Data shown in A-B represent three independent experiments, C-F represent two independent experiments. Data were shown as mean ± SEM by one-way ANOVA and Tukey's HSD post hoc analysis. *p<0.05, **p<0.01, ***p<0.001, ****, p<0.0001.

Figure S4

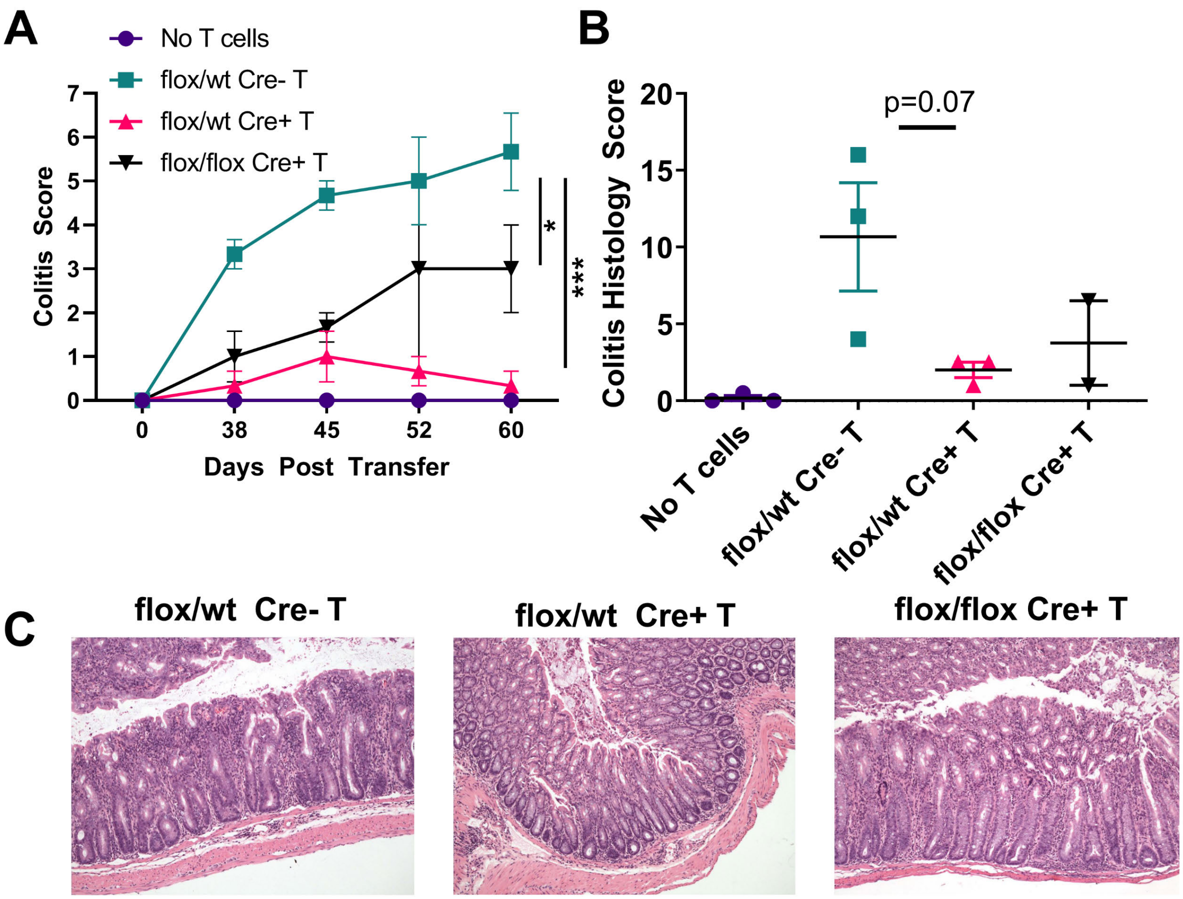

Figure S4. Fli-1 regulates T-cell pathogenicity in murine colitis. Rag1-/- deficient hosts on a B6 background were transplanted with 1 x 10^6 naïve $CD4^+$ T cells (CD8-CD25-CD44-CD62L+) isolated from *Fli1*$^{wt/wt}$, *Fli1*$^{flox/wt}$, or *Fli1*$^{flox/flox}$ donors. Colitis clinical score **(A)** and pathologic scores of H&E stained colon sections were analyzed from mice 60 days after T-cell transfer **(B)**. Representative images of colon H&E histology from each indicated group shown at 10x magnification **(C)**. Data represent an individual experiment (No T-cells n=3; Cre- T n=3; flox/wt T n=3; flox/flox T n=3). Significance was determined by using mixed model tests for clinical scores, and one-way ANOVA for other data. *p<0.05, **p<0.01, ***p<0.001.

Figure S5

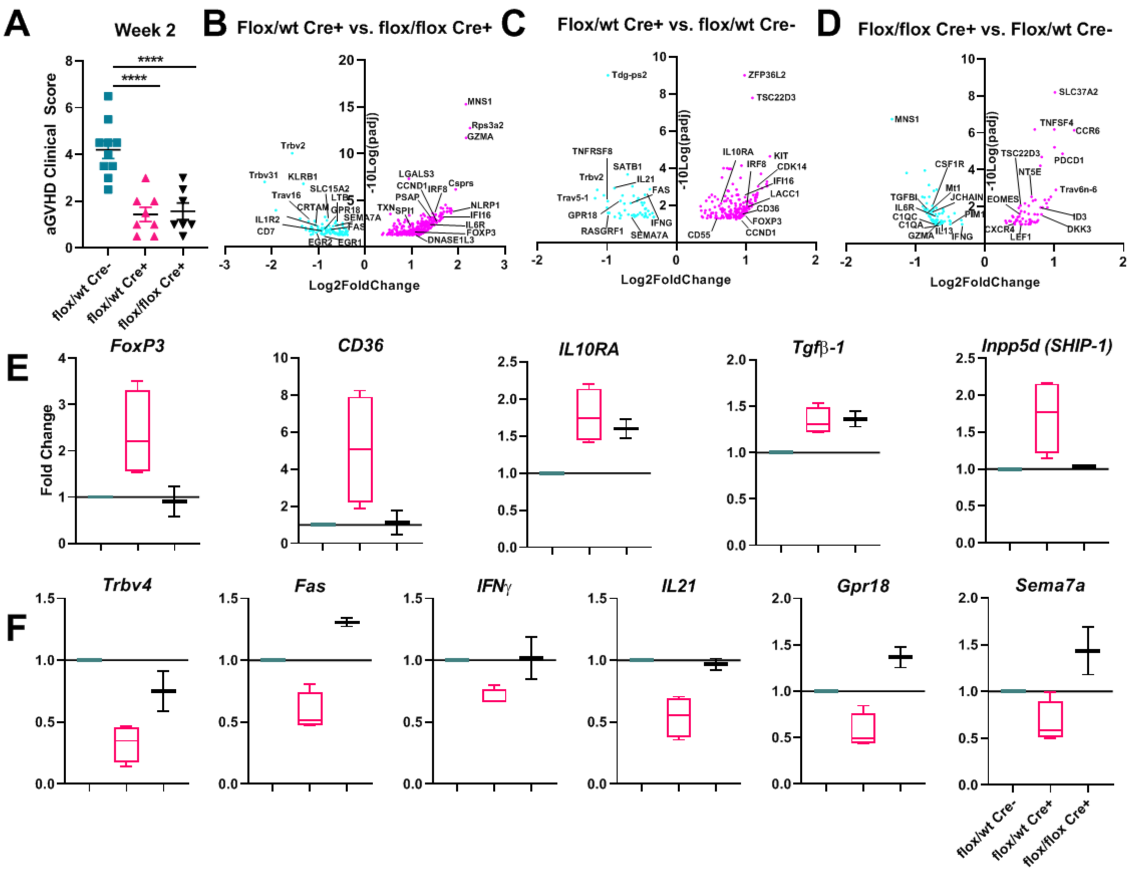

Figure S5. Fli-1 contributes to regulation of genes involved in Treg and effector T-cell development and function. aGVHD BMT was performed using 0.5 x 10^6 *Fli1*^{WT}, *Fli1*$^{flox/wt}$, or *Fli1*$^{flox/flox}$ purified donor total T cells and 5 x 10^6 donor TCD-BM from CD45.1 C57BL/6 mice. Week 2 after aGVHD BMT cumulative clinical scoring data **(A)**. On day 14 after aGVHD BMT, total $CD4^+$ and $CD8^+$ cells were isolated from spleens of BMT recipients via positive selection. Total mRNA was extracted from purified T cells and subjected to RNAseq analysis. Volcano plots of the most differentially expressed genes (DEG) between *Fli1* heterozygous (flox/wt Cre+), and *Fli1* homozygous (flox/flox Cre+) **(B)**, between flox/wt Cre+ and wild-type (flox/wt Cre-) **(C)**, between flox/flox Cre+ and flox/wt Cre- groups from RNAseq data **(D)**. Five of the significantly upregulated DEG **(E)** and six of the significantly downregulated DEG **(F)** were confirmed via qRT-PCR and are shown as fold change over *Fli1*^{WT} after normalization to *β-Actin* and *18sRNA* housekeeping genes. aGVHD scores in **A** represent three independent experiments. RNAseq was performed on one of three sets of mice from **A** where sample sizes were as follows (*Fli1*^{WT} n=4; *Fli1*$^{flox/wt}$ n=4; *Fli1*$^{flox/flox}$ n=2). Differential expression analysis between two conditions/groups (two biological replicates per condition) was performed using the DESeq2 R package.

Figure S6

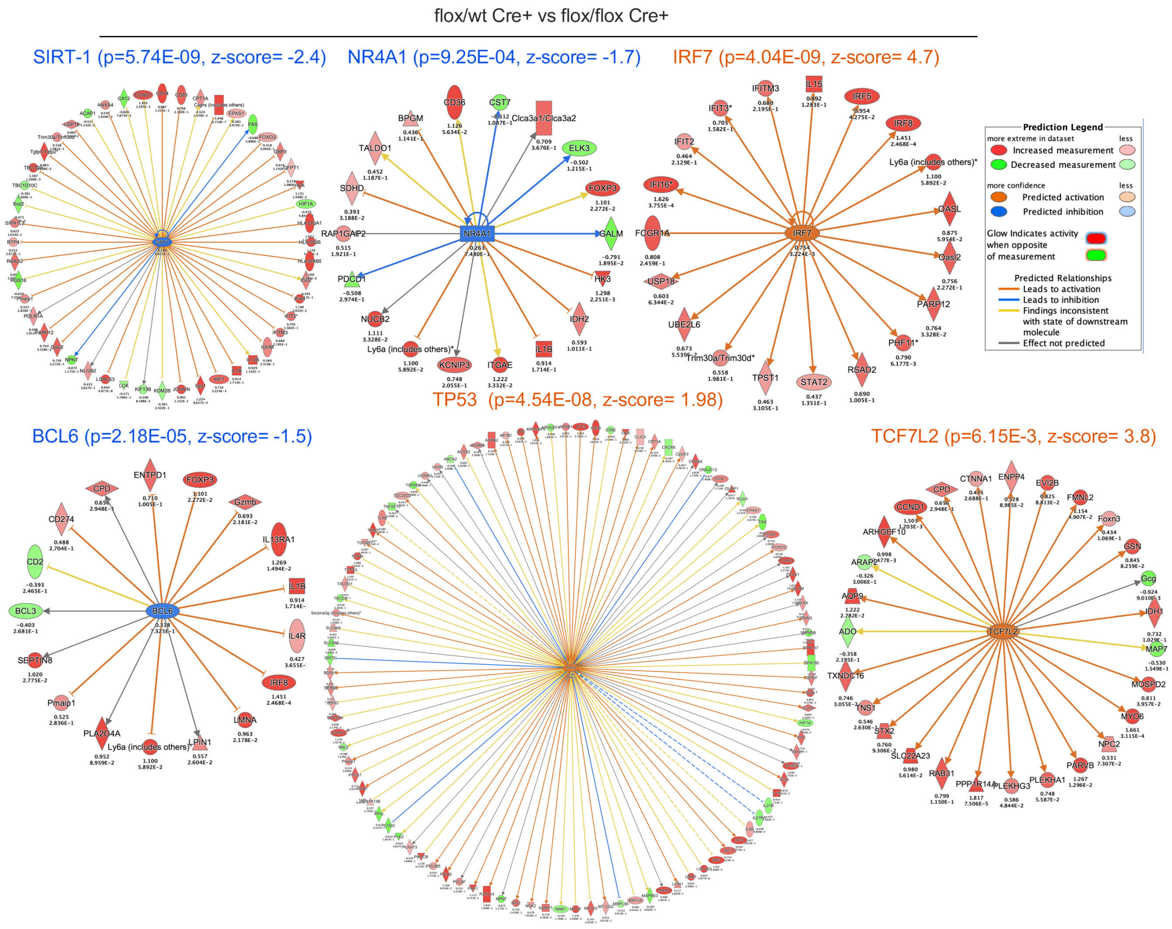

Figure S6. Predicted differential upstream regulator pathways between Fli-1 flox/wt Cre+ and flox/flox Cre+ T cells during GVHD. RNAseq expression data, collected as in Fig. S5, was used to calculate predictions of upstream targets that could be regulating observed transcriptomic differences between *Fli1 flox/wt* Cre+ and *flox/flox* Cre+ groups. Upstream regulator pathway labels are shown together with the significance of the prediction (p-value) and the activation z-score above each pathway (blue indicates pathway inhibition and orange indicates pathway activation) **(A)**. Prediction legend for each pathway is shown (top right). Log2Foldchange (top) and p(adj) value (bottom) are shown for each individual gene within the pathway.

Figure S7

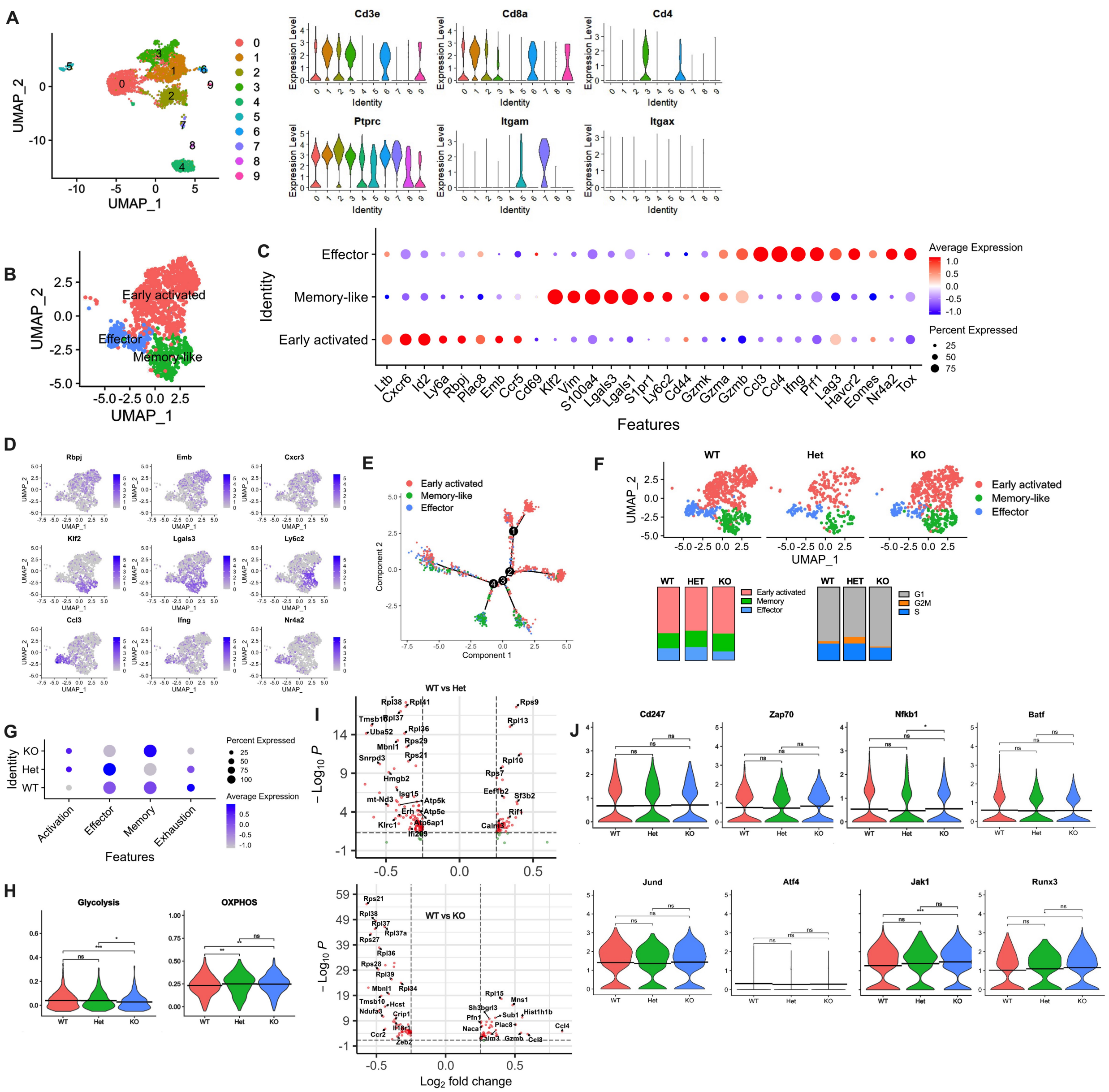

Figure S7. Fli-1 regulates gene transcription involved in activation, differentiation and function of CD8 T cells. (B) Integrated UMAP showing three major CD8 T-cell clusters isolated from the spleen of BALB/c recipients that were transplanted with BM (Rag1$^{-/-}$) and T cells from *Fli1*$^{wt/wt}$, *Fli1*$^{flox/wt}$, or *Fli1*$^{flox/flox}$ donors on day 14. **(C)** Expression of cell-defining features across all cell types. Color intensity is proportional to the average gene expression across cells in the indicated clusters. The size of circles is proportional to the percentage of cells expressing the indicated genes. **(D)** mRNA expression of the indicated genes projected on the UMAP in three subpopulations of cells. **(E)** Single cell trajectory of total CD8^{+} T cell subsets. **(F)** Integrated UMAP shows *Fli1*^{WT}, *Fli1*^{Het} and *Fli1*^{KO} CD8 T-cell clusters separately. Histogram shows frequency of each cell type cluster (left) and frequency of cells in each cell-cycle phase (right) in *Fli1*^{WT}, *Fli1*^{Het} and *Fli1*^{KO} CD8 T cells. **(G)** Dot plot shows activation, effector, memory, and exhaustion gene module scores in CD8 T cells. **(H)** Violin plot shows glycolysis and OXPHOS gene module scores in CD8 T cells. **(I)** Volcano plots of the most differentially expressed genes (DEG) between *Fli1*^{WT} versus and *Fli1*^{Het} (top) and *Fli1*^{WT} versus and *Fli1*^{KO} (bottom) CD8 T cells.

(J) Violin plot shows indicated gene expression in CD8 T cells. Significance was determined by using one-way ANOVA. *$p<0.05$, **$p<0.01$, ***$p<0.001$.

Figure S8

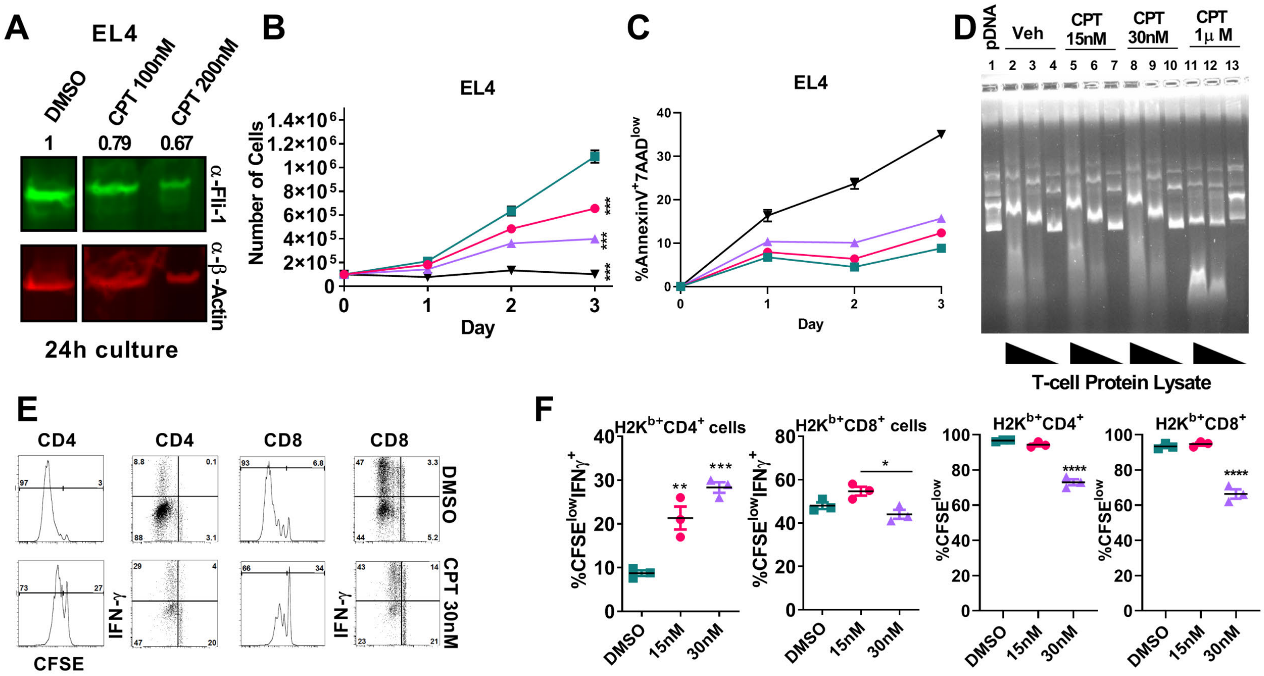

Figure S8. Low-dose camptothecin (CPT) inhibits Fli-1, reduces tumor growth and murine T-cell proliferation. Murine tumor line EL4 (T-cell leukemia) was treated with DMSO or indicated doses of CPT for 24 h, and pooled total cell lysates from at least 5 replicate wells were extracted for western blot of Fli-1 and β-Actin loading control protein expression, data represent two similar experiments **(A)**. Cell growth **(B)** and apoptosis **(C)** was quantified via flow cytometry using Annexin V and 7-AAD staining. **(D)**Topoisomerase I enzyme assay: Primary murine wild-type T cells were activated via polyclonal stimulation in the presence of vehicle, 15nM, 30nM, or 1μM CPT for ~48h. On day 2, total cell lysates were extracted from cultured T cells and subjected to topoisomerase I assay as described below in "supplementary materials and methods" section. Gel loading order: L1= plasmid DNA; L2-4=T cells + vehicle; L5-7= T cells + 15nM CPT; L8-10= T cells + 30nM CPT; L11-13= T cells + 1μM CPT. The amount of T-cell protein lysate added into the plasmid DNA Top I enzyme reaction was titrated down from left to right (5μg, 2.5μg, 0.5μg total protein) within each condition. Murine primary T cells from wild-type B6 mice were CFSE labeled and activated *in vitro* via polyclonal stimulation for ~72h in the presence of vehicle or 15nM to 30nM CPT. Representative flow cytometry plots show cell proliferation and intracellular IFN-γ production **(E)** and frequencies of proliferated and IFN-γ producing cells **(F)**. Data in B-F represent two independent experiments, each run in triplicate for each condition. Significance was determined by using one-way ANOVA. *p<0.05, **p<0.01, ***p<0.001.

Figure S9

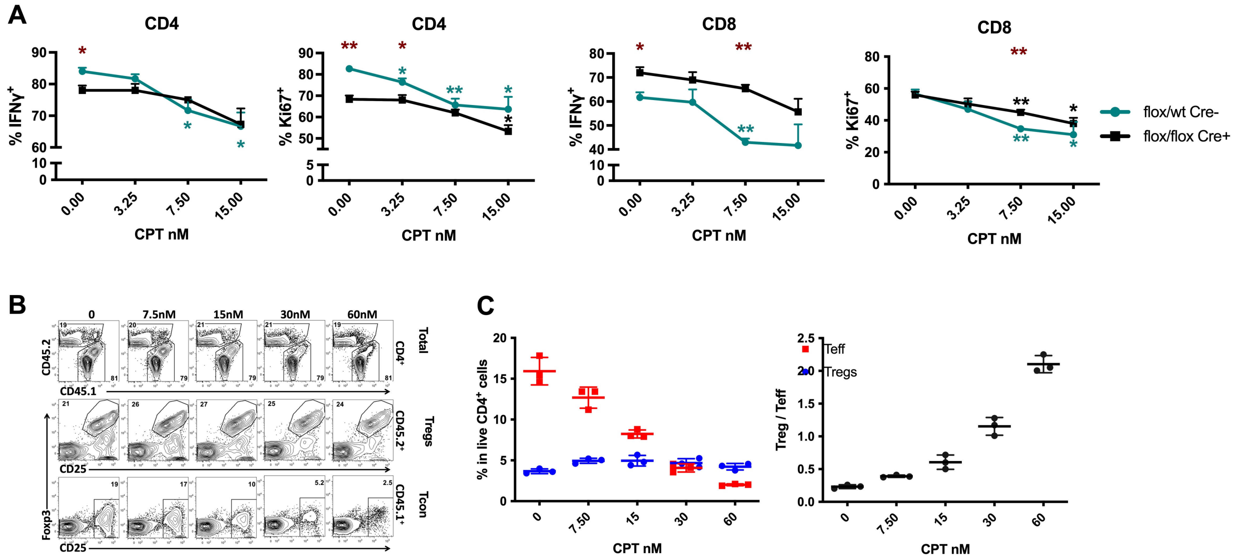

Figure S9. Low-dose CPT inhibits activation of WT T cells and suppresses effector T cells while sparing Tregs in vitro. Purified T cells isolated from *Fli1*^{flox}/^{WT} Cre- (WT) or *Fli1*$^{flox/flox}$ Cre+ (*Fli1* KO) were stimulated with plate bounded anti-CD3 and soluble anti-CD28 with CPT treatment at different concentration. On day 4, cells were analyzed for ki67 and IFNγ expression in gated live CD4 and CD8 T cells **(A)**. Statistical analysis of difference between indicated concentrations of CPT vs no CTP treated WT (green), *Fli1* KO T cells (black), or WT vs *Fli1* KO T cells (red) are shown **(A)**. CD25^{+}CD4^{+} Tregs isolated from CD45.2^{+} Foxp3^{GFP} mice were co-cultured with CD25^{-}CD4^{+} conventional T cells isolated from CD45.1^{+} B6 mice together with allogeneic APCs from FVB mice. The cells were cultured with different concentrations of CPT for 4 days. The frequencies of Tregs (CD45.2^{+}GFP^{+}CD25^{+}) and effectors T cells (CD45.1^{+}CD25^{+}) within live CD4 T cells, and the ratio of Tregs to effector T cells are shown **(B-C)**. Data represent two independent experiments, each condition was run in triplicate. Significance was determined by using unpaired 2-tailed Student *t* test. *p<0.05, **p<0.01, ***p<0.001.

Figure S10

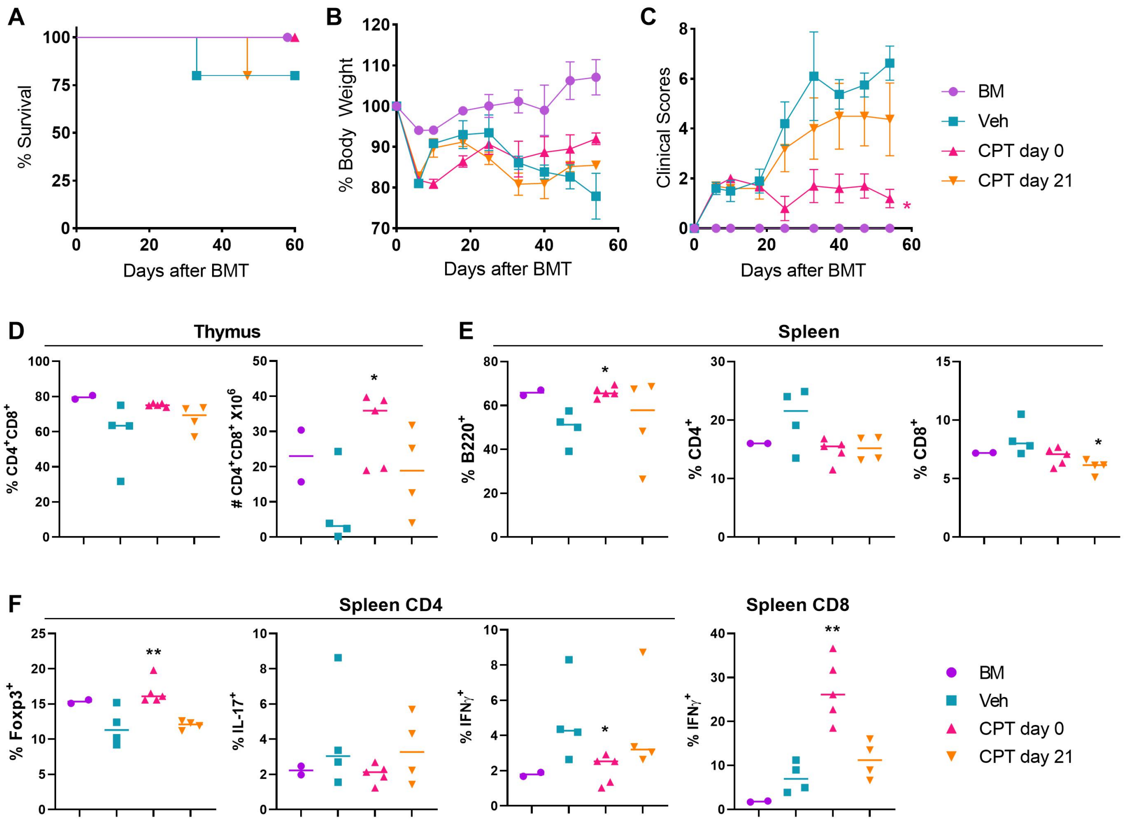

Figure S10. Low-dose camptothecin (CPT) effectively prevent cGVHD development after MHC-matched BMT. Lethally irradiated BALB/c mice were transplanted with 5×10^6 TCD-BM cells supplemented with or without 5×10^6 splenocytes from B10.D2 donors. The recipient mice were i.p. injected with CPT at 0.25mg/kg every other day for 2 weeks starting on day 0 or day 21 after BMT. Recipient survival **(A)**, body weight change **(B)** and clinical score **(C)** were monitored following BMT. On day 60, recipient spleen and thymus were harvested for flow analysis. Frequencies and absolute numbers of CD4$^+$CD8$^+$ thymocytes gated on live donor-derived (Ly9.1$^-$) cells are shown **(D)**. Frequencies of B220$^+$, CD4$^+$ and CD8$^+$ cells gated on live donor-derived cells are shown in recipient spleen **(E)**. Frequencies of Foxp3$^+$, IL-17$^+$ and IFNγ$^+$ cells in gated live donor-derived CD4 T cells, and frequencies of IFNγ$^+$ cells in gated live donor CD8 T cells in recipient spleen **(F)**. BM n=2; VEH n=5; CPT day 0 n=5; CPT day 21 n=5. Significance was determined by using mixed model tests for clinical scores and body weight, log-rank test for survival data, and one-way ANOVA for all other data. *p<0.05, **p<0.01.

Figure S11

Figure S11. Etoposide inhibits Fli-1 in activated human PBMC and Jurkat cells. Jurkat cells were

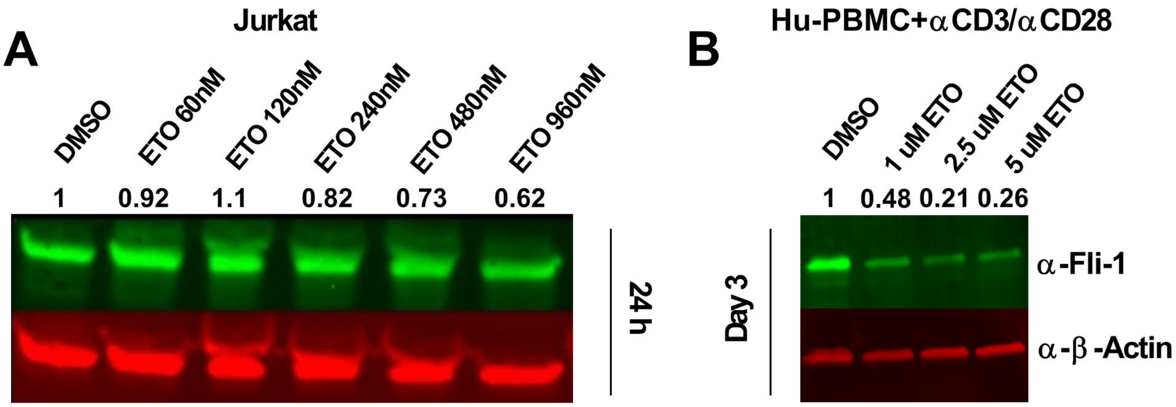

treated with the indicated concentrations of etoposide (ETO) for ~24h. At ~24h, total cell lysates were extracted for western blot of Fli-1 and β-actin proteins **(A)**. Total PBMC from a healthy donor was stimulated *in vitro* with soluble anti-CD3 (2 µg/mL) and anti-CD28 (1 µg/mL) for 3 days with or without DMSO or the indicated concentrations of Etoposide. On day 3, total cell lysates were extracted for western blot of Fli-1 and β-actin proteins **(B)**. Data in A and B are from independent blots.

Figure S12

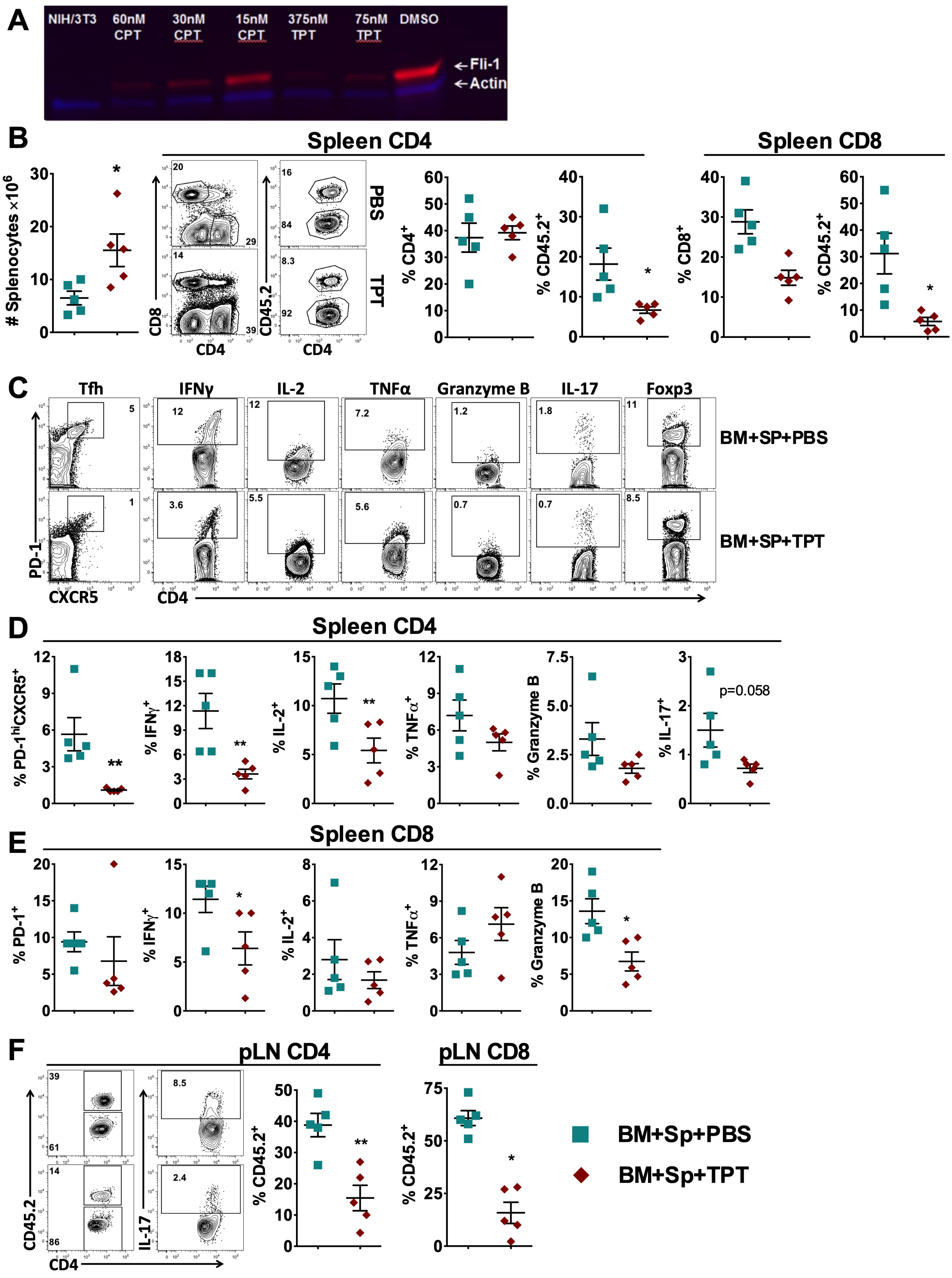
A
NIH/3T3
60nM CPT
30nM CPT
15nM CPT
375nM TPT
75nM TPT
DMSO
←Fli-1
←Actin
B
Spleen CD4
Spleen CD8
Splenocytes ×10^6
PBS
TPT
CD8
CD4
CD45.2
% CD4+
% CD45.2+
% CD8+
C
Tfh
IFNγ
IL-2
TNFα
Granzyme B
IL-17
Foxp3
BM+SP+PBS
BM+SP+TPT
PD-1
CXCR5
CD4
D
Spleen CD4
% PD-1hiCXCR5+
% IFNγ+
% IL-2+
% TNFα+
% Granzyme B
% IL-17+
p=0.058
E
Spleen CD8
% PD-1+
% IFNγ+
% IL-2+
% TNFα+
% Granzyme B
F
pLN CD4
pLN CD8
CD45.2
IL-17
CD4
% CD45.2+
BM+Sp+PBS
BM+Sp+TPT

Figure S12. Topotecan inhibits T-cell allogeneic response. Jurkat cells were treated with the indicated concentrations of CPT or Topotecan (TPT) for ~24h. At ~24h, total cell lysates were extracted for western blot of Fli-1 and β-actin proteins **(A)**. Splenocytes ($CD45.2^+$) from B6 mice were injected together with TCD-BM ($CD45.1^+$) into lethally irradiated BALB/c mice. The recipient mice were i.p. injected with topotecan (TPT) at 0.3mg/kg every other day starting on the day of BMT for 10 days. Absolute cell number of recipient splenocytes, frequencies of CD4 and CD8 T cells within total donor cells ($H2K^{b+}$) and frequencies of $CD45.2^+$ cells within donor CD4 or CD8 T cells are shown on day 60 post-BMT **(B)**. Representative flow figures and bar graphs show the frequencies of Tfh ($PD\text{-}1^+CXCR5^+$) and cytokine producing cells in gated CD4 or CD8 donor T cells in recipient spleen **(C-E)**. Frequencies of $CD45.2^+$ cells within donor CD4 or CD8 T cells and flow figures of IL-17 producing cells in donor CD4 T cells are show in recipient pLNs **(F)**. Significance was determined by using an unpaired 2-tailed Student *t* test. *p<0.05, **p<0.01, ***p<0.001.

Figure S13

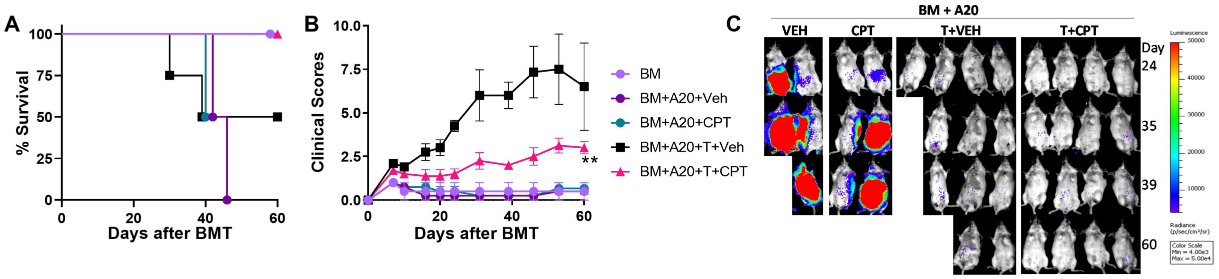

Figure S13. Camptothecin (CPT) treatment preserves T-cell mediated GVL activity. Lethally irradiated BALB/c mice were transplanted with 5×10^6 TCD-BM cells supplemented with or without 1×10^6 purified total T cells from B6 donors. Four groups of the recipients were also supplemented with 2,000 A20 at the time of BMT, and treated with vehicle, CPT 0.25mg/kg only, mature T cells plus vehicle, or mature T cells plus CPT 0.25mg/kg on day 0 every other day until day 14 post-BMT. Recipient survival **(A)**, clinical score **(B)** and A20 tumor growth **(C)** were monitored after BMT. BM n=2; BM+A20+VEH n=2; BM+A20+CPT n=2; BM+A20+T+VEH n=4; BM+A20+T+CPT n=4. Significance was determined by using mixed model tests for clinical scores and log-rank test for survival data. **p<0.01.

Figure S14

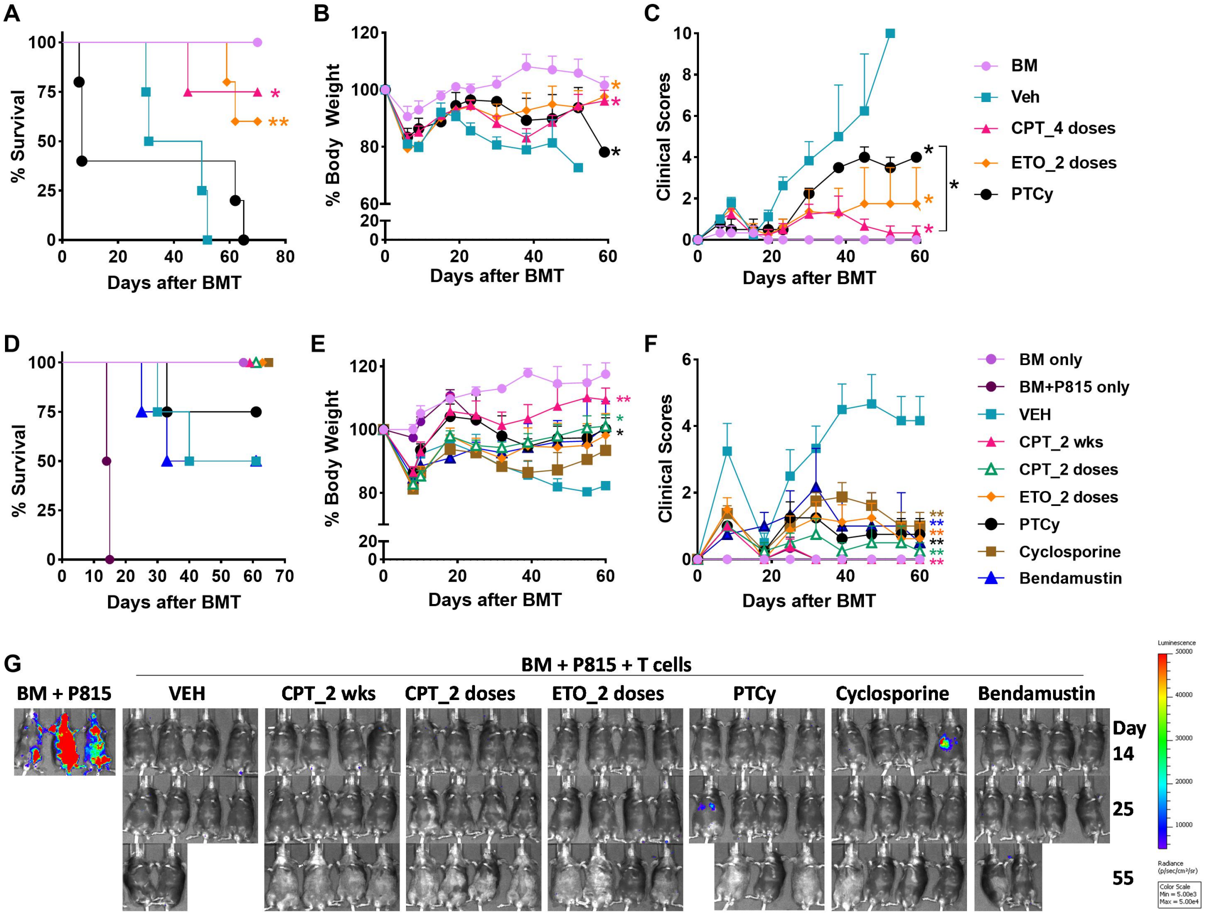

Figure S14. Short-term inhibition Fli-1 with Camptothecin (CPT) or Etoposide (ETO) effectively controls GVHD whiles sparing GVL activity. **(A-C)** Lethally irradiated B6D2F1 mice were transplanted with TCD-BM cells supplemented with or without purified $CD25^-$ T cells from B6 donors. Recipients were treated with vehicle or CPT 0.25mg/kg on day 0, 2, 4 and 6 after BMT. ETO or cyclophosphamide was administrated at 5mg/kg and 50mg/kg, respectively, on day 3 and 4 after BMT. Recipient survival **(A)**, body weight **(B)** and clinical scores **(C)** were monitored after BMT. (BM n=3; VEH n=4; CPT_4 doses=4; ETO_2 doses n=5; PTCy n=5), *p<0.05, **p<0.01. **(D-G)** In a separate experiment, recipients were i.v. injected with 5,000 luciferase-transduced P815 cells on the day of BMT. Recipients were i.p. injected with vehicle, CPT 0.25mg/kg on day 0 administered every other day until day 14 post-BMT, or CPT, ETO or cyclophosphamide at 0.5mg/kg, 5mg/kg and 12.5mg/kg, respectively, on day 3 and 4 after BMT. In addition, recipients were i.p. injected with cyclosporine 20mg/kg administrated daily or bendamustin 30mg/kg administrated on day 3 after BMT. Recipient survival **(G)**, clinical score **(H)** and P815 tumor growth **(I)** were monitored following BMT. BM only n=3; BM+P815 n=3; VEH=4; CPT_2 wks n=4; CPT_2 doses n=4, ETO_2 doses n=4; PTCy n=4; cyclosporine n=4; bendamustin n=4. Significance was determined by using mixed model tests for clinical scores and body weight and log-rank test for survival data. *p<0.05, **p<0.01.

Figure S15

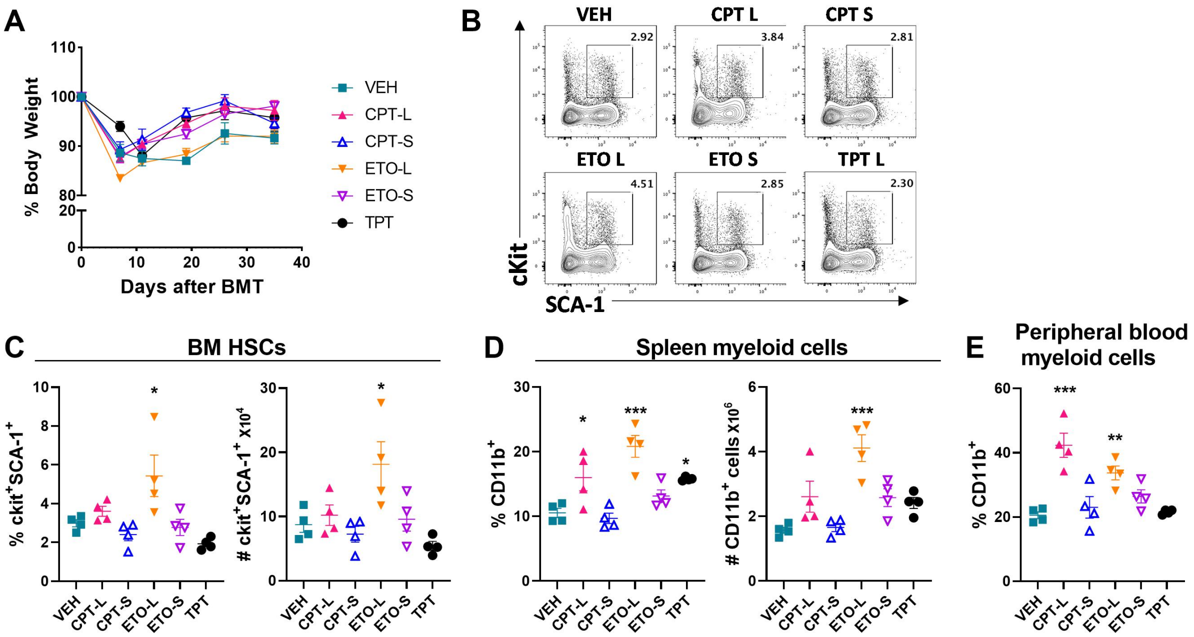

Figure S15. Inhibition of Fli-1 with Camptothecin (CPT), Etoposide (ETO) or Topotecan (TPT) did not adversely impact hematopoietic stem cells (HSCs) and myeloid cells. Lethally irradiated BALB/c mice were transplanted with 5 x 10^6 TCD-BM cells supplemented from B6 donors. Recipient were treated with CPT, ETO or TPT at 0.25mg/kg, 5 mg/kg, or 0.3mg/kg, respectively, on day 0 every other day until day 14 post-BMT (CPT-L, ETO-L and TPT). For the short-term treatment, CPT or ETO was administrated at 0.5mg/kg and 5mg/kg, respectively, on day 3 and 4 post BMT (CPT-S and ETO-S). Recipient body weight change was monitored following BMT **(A)**. Frequency of donor derived HSCs in gated live $H2K^{b+}lin^-$ and their absolute numbers are shown in recipient BM from femur on day 35 after BMT **(B-C)**. Frequency and number of donor myeloid cells ($CD11b^+$) in gated live $H2K^{b+}$ cells are shown in recipient spleen and peripheral blood **(D-E).** Significance was determined by using mixed model tests for body weight and one-way ANOVA for all other data. *p<0.05, **p<0.01.

Figure S16

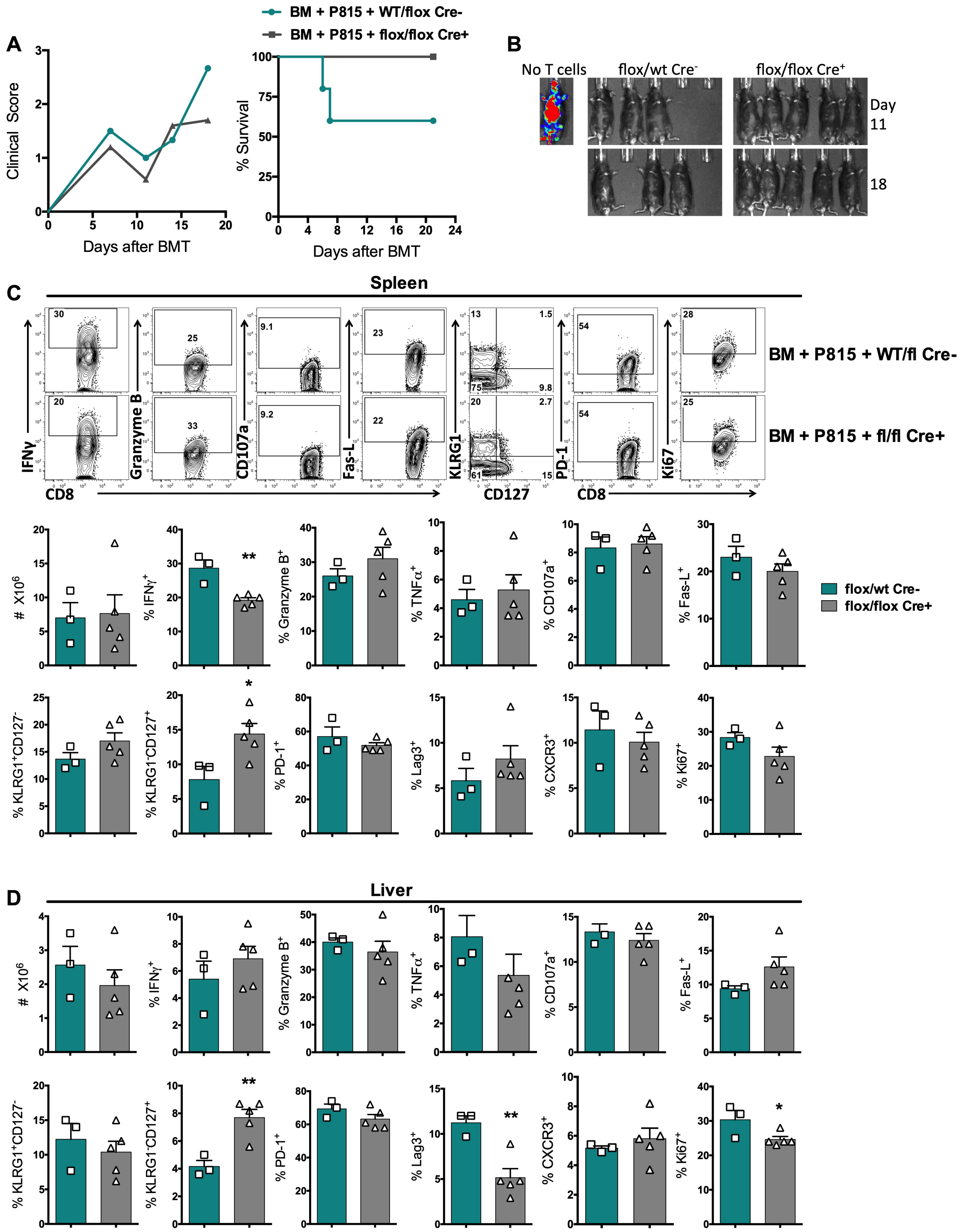

Figure S16. Fli-1 expression in donor T cells is not essential for GVL response and CD8 T-cell function. Purified T cells isolated from the donor mice with indicated genotype plus Rag1$^{-/-}$ BM were transferred into lethally irradiated B6D2F1 mice. On the day of BMT, 5,000 luciferase-transduced P815 cells were i.v. injected into these recipients. Clinical scores and survival were monitored following BMT **(A)**. IVIS2000 imager was used to periodically monitor firefly-luciferase expression of transplanted P815 cells in recipient mice injected with D-luciferin substrate at each imaging time-point **(B)**. Absolute cell

numbers of splenocytes and lymphocytes in liver, and frequencies of IFNγ, granzyme B, TNFα, CD107a, Fas-L, KLRG1, CD127, PD-1, Lags, CXCR3 and ki67 expression are shown in gated donor CD8 T cells in spleen **(C)** and liver **(D)** on day 21 post-BMT. Significance was determined by using an unpaired 2-tailed Student *t* test. *p<0.05, **p<0.01, ***p<0.001.

Figure S17

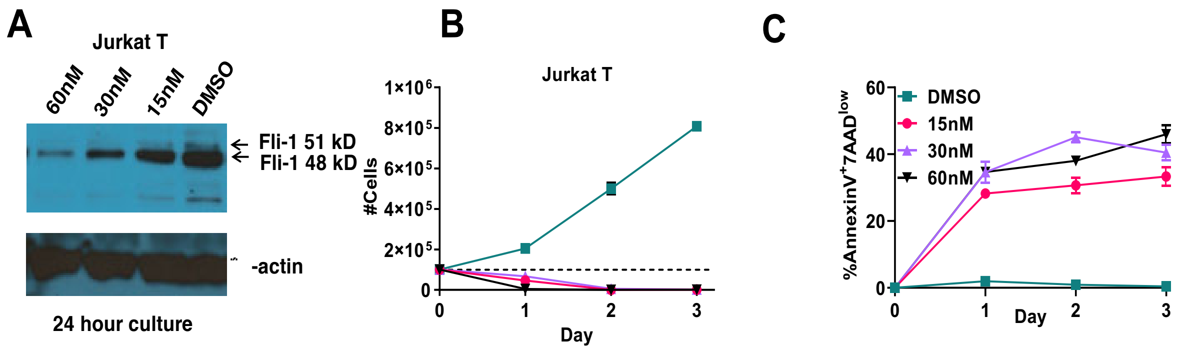

Figure S17. Camptothecin inhibits Fli-1 expression and growth in human Jurkat cells. Human Jurkat cells were cultured with vehicle or 15nM to 60nM camptothecin (CPT) for ~24 hours. At 24h, total cell lysates were obtained from Jurkat cultures and blotted for Fli-1 and β-actin **(A)**. Jurkat cell growth **(B)** and apoptosis (Annexin V+7AAD^{low}) **(C)** was measured via flow cytometry at the indicated time points.

Figure S18

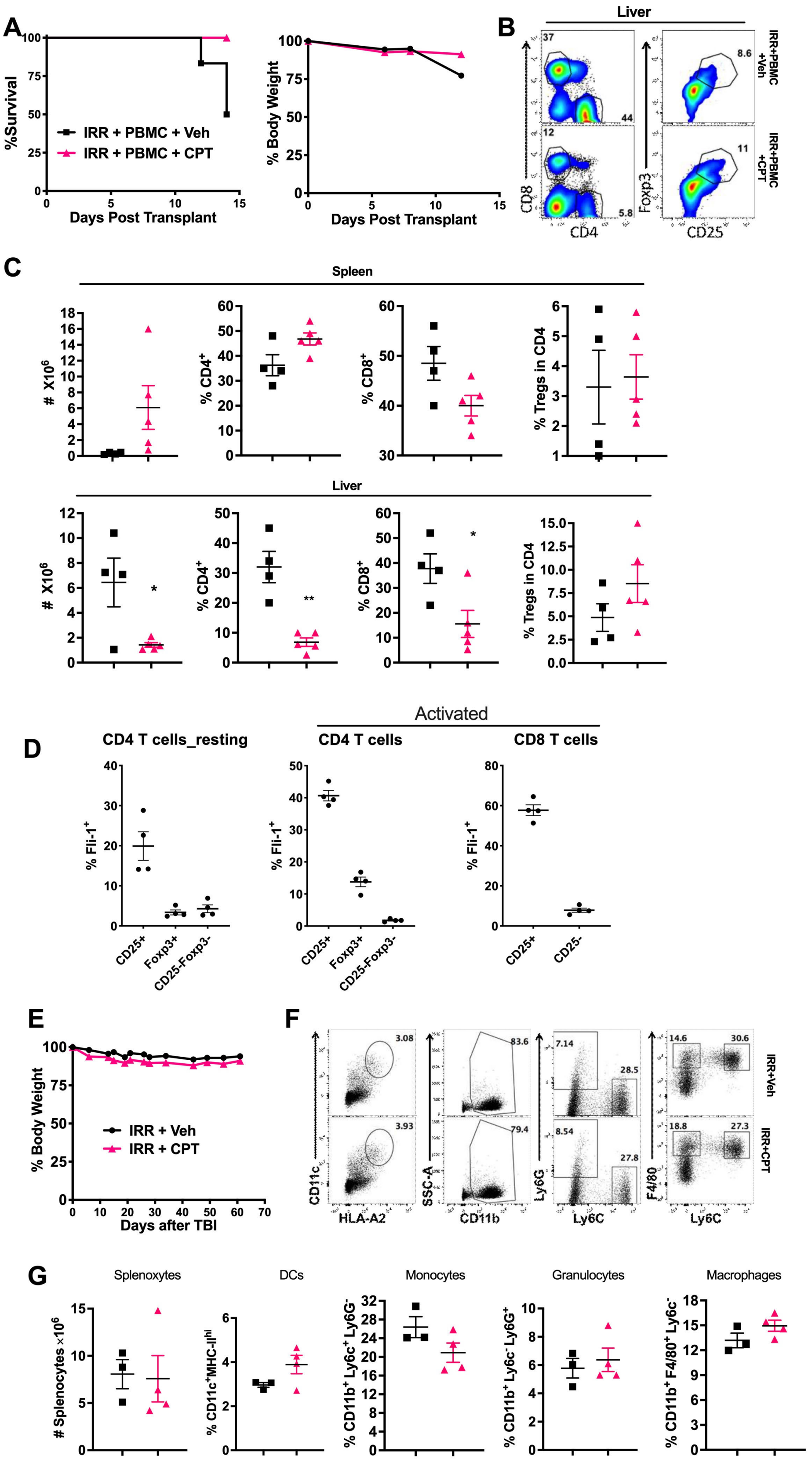

A
%Survival
Days Post Transplant
IRR + PBMC + Veh
IRR + PBMC + CPT
% Body Weight
B
Liver
IRR+PBMC +Veh
IRR+PBMC +CPT
CD8
CD4
Foxp3
CD25
C
Spleen
Liver
X10^6
% CD4+
% CD8+
% Tregs in CD4
D
CD4 T cells_resting
Activated
CD4 T cells
CD8 T cells
% Fli-1+
CD25+
Foxp3+
CD25-Foxp3-
CD25-
E
% Body Weight
Days after TBI
IRR + Veh
IRR + CPT
F
IRR+Veh
IRR+CPT
CD11c
HLA-A2
SSC-A
CD11b
Ly6G
Ly6C
F4/80
G
Splenoxytes
DCs
Monocytes
Granulocytes
Macrophages
Splenocytes ×10^6
% CD11c+MHC-IIhi
% CD11b+ Ly6c+ Ly6G-
% CD11b+ Ly6c- Ly6G+
% CD11b+ F4/80+ Ly6c-

Figure S18. CPT treatment had little impacts on Treg and myeloid cell populations. HLA-A2^{+} NSG mice were sub-lethally irradiated (250cGy) and transplanted with 8 to 10 x 10^{6} total human PBMC from healthy donor (HLA-A2-) to induce human GVHD. These mice received vehicle or CPT 0.25-0.5mg/kg on day 0 administered every other day and recipient survival **(A)** and body weight was monitored for 2 weeks. Absolute cell numbers, frequencies of CD4, CD8 and Foxp3^{+} cells were analyzed in recipient spleen and liver **(B-C)**. Human PBMC were cultured in 10% FBS 1640 RPMI medium and supplemented with 1ug/ml anti-CD3 and tested Fli-1 expression by flow cytometry on day 0 and 3 **(D).** HLA-A2^{+} NSG mice were irradiated at 250cGy followed by i.p. injection of vehicle control or CPT at 0.25mg/kg every other day for 2 weeks. Body weight **(E)** and absolute cell numbers of splenocytes, frequency of myeloid cell populations were analyzed on day 60 after irradiation **(F-G)**. Significance was determined by using an unpaired 2-tailed Student *t* test for comparing two groups and one-way ANOVA for comparing more than two groups. *p<0.05, **p<0.01, ***p<0.001, ****p<0.0001.

Table S1

Table 1: Primers Used in the Study			
**Genomic PCR (genotyping)**	**Accession Number**		
**Sequence Name**		**Base**	**Sequence (5'-->3')**
EcoRUp (Fli-1 Flox)	N/A	22	TAG TGA CTC AGC CTT AAC TCT C
EcoRDown (Fli-1 Flox)	N/A	18	TTG CAC TGA GAC GAT GTA
CD4 Cre_F	N/A	26	GGC TCA GAT TCC CAA CCA ACA AGA GC
CD4 Cre_R	N/A	23	CCT GGC GAT CCC TGA ACA TGT CC
**qRT-PCR**			
Fli-1_F	NM_008026.5	24	ACT TGG CCA AAT GGA CGG GAC TAT
Fli-1_R	NM_008026.5	18	CCC GTA GTC AGG ACT CCCG
Sema7a_F	NM_011352.3	20	ACG TGG CAA GGT CTA CCA CT
Sema7a_R	NM_011352.3	21	CCA CAG TCC TGT TTG TCC TGA
IL-21 (IDT DNA PrimeTime Assay: Mm.PT.58.7853071)	NM_021782	N/A	
PrimeTime Primer 1	NM_021782	21	GGT TTG ATG GCT TGA GTT TGG
PrimeTime Primer 2	NM_021782	24	TGA CTT GGA TCC TGA ACT TCT ATC
Fas (IDT DNA PrimeTime Assay: Mm.PT.58.41299055)	NM_007987	N/A	
PrimeTime Primer 1	NM_007987	21	GTT TGT ATT GCT GGT TGC TGT
PrimeTime Primer 2	NM_007987	21	ACC AGA CTT CTA CTG CGA TTC
GPR18 F	NM_182806.2	21	CAA TTA CCT TCG CAG TGT TCG
GPR18 R	NM_182806.2	22	GGT TCA CCC GAA AGT AGA TAG G
IL-10RA _F	NM_001324486.1	20	TGT GAC CAA CCT GAG CAT CT
IL-10RA_R	NM_001324486.1	21	TTG AAG ACC AGG ACT GTA GGC
Trbv4_F	NC_000072.6	21	GAA ACA GTT CCA AGG CGC TTC
Trbv4_R	NC_000072.6	21	GGC ACA GAG ATA CAC AGC AGA
CD36_F	NM_001159555.1	20	GGT GGA TGG TTT CCT AGC CT
CD36_R	NM_001159555.1	20	GGC CCG GTT CTA ATT CAT GC
FoxP3_F	NM_001199347.1	21	GCC ACT CCA GAC AGA AGA AAG
FoxP3_R	NM_001199347.1	20	CCA AGT CTC GTC TGA AGG CA

Supplementary Materials and Methods

Allogeneic bone marrow transplantation (Allo-BMT). In B6 to BALB/c cGVHD model, recipients were lethally irradiated (700cGy, X-ray source from Precision X320) on day -1 or 0, and infused with allogeneic B6 unfractionated splenocytes (0.5 x 10^6) from either *Fli-1*$^{wt/wt}$, *Fli-1*$^{fl/wt}$Cre+, or *Fli-1*$^{fl/fl}$Cre+ donors and supplemented with T-cell depleted bone marrow (TCD-BM) (5 x 10^6) from each respective strain (or from B6.Ly5.1$^+$ donors in certain experiments) and injected I.V. within 24h after irradiation. TCD-BM protocol was described in detail previously (1, 2). BALB/c recipients were monitored weekly with the cGVHD clinical scoring system described previously (2). On days 40-60 post-transplant, recipients were sacrificed and spleens, peripheral lymph nodes, and thymus were collected for flow cytometry analysis, and GVHD target organs were paraformaldehyde fixed and sectioned for hematoxylin and eosin (H&E) staining. In B6 to BALB/c aGVHD model, conditions were similar, except instead of total splenocytes, a dose of 0.5 x 10^6 total T cells was infused and all recipients were given B6.Ly5.1$^+$ TCD-BM. An independent pathologist scored histology sections for cGVHD, aGVHD, and colitis as described previously (2). B6 to B6D2F1 acute GVHD and GVL model using P815 mastocytoma cells was also used for pharmacological studies and was described previously (3). The recipient mice were age, sex, and vendor matched in the same batch for any given experiment throughout the study. For donor mice, we used age and sex matched littermates with different genotypes for each experiment in the study.

Graft-versus-leukemia (GVL) response. Wild-type B6 donor grafts were given to lethally irradiated B6D2F1 recipients with or without 5,000 P815 cells per mouse together with TCD-BM (5 × 10^6 per mouse) with or without 3 x 10^6 purified total T cells. Mice given TCD-BM plus P815 cells were treated with either vehicle (DMSO) or camptothecin (0.25mg/kg q.o.d) alone without donor T cells were used as controls. Recipients were subjected to bioluminescent imaging (IVIS200 System, Perkin Elmer) at periodic time points as shown following allo-BMT to assess P815 growth and relapse.

Colitis Model. Rag1$^{-/-}$ mice on a B6 background were given syngeneic transplant of 1 x 10^6 naïve $CD4^+$ T cells ($CD4^+CD8^-CD25^-CD44^-$) isolated from spleen and lymph nodes from either *Fli1*$^{wt/wt}$, *Fli1*$^{flox/wt}$, or *Fli1*$^{flox/flox}$ mice. Mice were followed weekly for colitis clinical score (described previously (4)) and on day 60 post T-cell transfer, mice were sacrificed and colonic sections were stained with H&E and scored for colitis via an independent pathologist using previously established criteria (5).

Treatment with Fli-1 inhibiting drugs camptothecin, etoposide and topotecan. Camptothecin (CPT), etoposide (ETO) and Topotecan (TPT) are known chemotherapeutics via their inhibition of topoisomerase enzymatic activity but have demonstrated potent off-target effects against Fli-1 protein level. CPT in particular can deplete Fli-1 protein on multiple different human and mouse cell lines at very low concentrations, shown in the current study and by other groups (6). Each drug in its powder form was dissolved in dimethyl sulfoxide (DMSO) at a concentration of 3mg/mL (CPT), 8mg/mL (ETO), or in PBS at a concentration of 60μg/ml (TPT) aliquoted and then stored at −80°C. For CPT, aliquots were brought to 0.1 mg/mL with DMSO, and animals were injected i.p. at 0.25-0.5mg/kg 2 to 4 hours prior to allo-BMT as prevention or on day 28-30 after BMT for treatment, and followed with every other day injections until days 14 or 28 (prevention) or from days 28-56 (treatment). For ETO, aliquots were brought to 0.8 mg/mL with a vehicle solution containing 30% PEG300 and sterile nanopure H_2O, and was injected i.p at 2 or 5mg/kg on day 0 and every other day until day 14. For TPT, aliquots were brought to 30μg/ml with sterile 1 x PBS and animals were injected i.p. at 0.3mg/kg on day 0 and every other day until day 10 after BMT.

qRT-PCR and RNAseq. Total RNA was extracted from cells using TRIzol reagent according to its protocol and converted to cDNA using iScript Reverse Transcription Supermix for RT-qPCR (Bio-Rad). qRT-PCR was carried out using ssoAdvanced Universal SYBR Green Supermix (Bio-Rad) and reactions were amplified and quantified on the Bio-Rad CFX96 instrument. qRT-PCR primers used are provided in Supplemental methods Table 1. RNA was further purified from TRIzol extracted samples using RNEasy

column purification (Qiagen) according to the company protocol and used for RNAseq carried out by Novogene (Sacramento, CA) according to their company protocol: RNAseq downstream analysis was performed using a combination of programs including STAR, HTseq, Cufflink and Novogene wrapped scripts. Alignments were parsed using Tophat program and differential expressions were determined through DESeq2/edgeR. Reference genome and gene model annotation files were downloaded from genome website browser (NCBI/UCSC/Ensembl) directly. Indexes of the reference genome was built using STAR and paired-end clean reads were aligned to the reference genome using STAR (v2.5). STAR used the method of Maximal Mappable Prefix (MMP) which can generate a precise mapping result for junction reads. (For DESeq2 with biological replicates) Differential expression analysis between two conditions/groups (two biological replicates per condition) was performed using the DESeq2 R package (2_1.6.3). DESeq2 provide statistical routines for determining differential expression in digital gene expression data using a model based on the negative binomial distribution. The resulting P-values were adjusted using the Benjamini and Hochberg's approach for controlling the False Discovery Rate(FDR). Genes with an adjusted P-value <0.05 found by DESeq2 were assigned as differentially expressed.

Topoisomerase I enzymatic activity assay. Primary wild-type mouse T cells were polyclonally activated via anti-CD3 (plate-bound 2μg/mL) and anti-CD28 (soluble 2μg/mL) and cultured with DMSO (vehicle) or low-doses (15nM, 30nM) or high doses (500nM, 1μM) of CPT for 48h. On day 2, whole-cell lysates were extracted from T-cell cultures, and these fresh lysates containing endogenous topoisomerases were incubated with Topoisomerase I reaction buffer and supercoiled plasmid DNA for 30 min at 37ºC. Reaction products were then run on 0.8% agarose gel to determine ability of endogenous topoisomerase I to relax supercoiled plasmid DNA as described previously (7).

Cell lines, Flow cytometry, ELISA, and Western Blotting. EL4 (TIB-39), Jurkat (TIB-152), P815 (TIB-64) and A20 (TIB-208) cell lines were originally purchased from ATCC (Manassas, Virginia). Splenocytes, thymocytes, and lymph node cells were analyzed for surface proteins and intracellular

cytokines using standard flow cytometric protocols, as previously described (1, 2). The following mouse antibodies were used for staining: Fixable Live/Dead yellow (BD Biosciences, San Jose, CA), anti-TCRβ–FITC or -PE–Cy7 (Clone: H57-597), anti-TCRvβ6-PE (RR4-7), anti-CD39-PE-Cy7 (Duha59), anti-cKit-FITC (D7), anti-SCA-1-APC (D7), human HLA-A2-biotin (BB7.2), anti-Ly6C-Pacific blue (HK1.4) from Biolegend (San Diego, CA), anti-CD4–FITC, or –V450 (RM4-5), anti-CD8α–FITC, or -allophycocyanin-cy7 (53-6.7), anti-PD-1–PE or –PerCpCy5.5 (J43), anti-CXCR5–PE-Cy7 (SPRCL5), anti-B220–V450 (RA3-6B2), anti-CD73-V450 (TY/23), anti-CD86–PE-Cy5 (GL1), anti-CD25-FITC (7D4), anti-IL-2-PE (JES6-5H4), anti-granzyme B-PE (NG2B), anti-TNFα-PE-Cy7 (MP6-XT22), anti-F4/80-BV711 (T45-2342), anti-Fas-L-biotin (MFL3), anti-Lag3-V450 (C9B7w), anti-CXCR3-biotin (CXCR3-173) and anti-ki67-PE (B56), anti-Ly6G-APC (1A8) purchased from BD Biosciences. anti–interferon-γ (IFN-γ) Per-cp5.5 (XMG1.2), IL-17A PE-Cy7or APC (eBio64DEC17), FoxP3 APC or PE (FJK-16s), anti-CD107a-APC (1D4B), anti-KLRG1-PE (2F1), anti-CD127-APC (A7R34), anti-CD11c-APC-Cy7 (N418), anti-CD11b-PE-cy7 (M1/70) from eBioscience. Cell isolates were analyzed using Diva software, LSR II (BD Biosciences), and FlowJo (TreeStar, Ashland, OR). Enzyme-linked immunosorbent assay (ELISA) using an IL-2 capture antibody (BD) and IL-2 biotin detection antibody (BD) was used to measure IL-2 from cell-culture supernatants. Primary rabbit anti-human/mouse Fli-1antibodies used for western blots were either produced in house from Dr. Xian Zhang or purchased from Abcam (cat# ab133485) and mouse anti-β-actin was purchased from Sigma Aldrich (cat# A2228). Secondary antibodies were either anti-rabbit (Santa Cruz, #sc2030) or anti-mouse (Southern Biotech, #1030-05) directly conjugated to HRP or to fluorescent markers IRDye680RD (LiCor, cat# 925-68070) or IRDye 800CW (LiCor, cat# 925-32211). Where indicated, the ratio of Fli-1 to β-Actin staining intensity was quantified and normalized to control samples using LI-COR Image Studio v. 5.2 software.

Single cell RNA sequence. Donor T cells were MACS-sorted using Thy1.2-beads from the recipient spleens 14 days after transplantation. The Single-cell RNA-seq (scRNA-seq) libraries were generated from those isolated T cells using Chromium Single Cell 3′ v.2. Libraries and were then loaded on the

Chromium Controller (10x Genomics). The libraries were sequenced using the HighSeq 4000 platform (Illumina) to a depth of about 300 million reads per library with 2 × 50 read length. Cell Ranger (10x Genomics) functions mkfastq and count were used to demultiplex the sequencing data and generate gene-barcode matrices, respectively. All scRNA-seq analyses were performed in R (version 4.1.2) using the package Seurat (version 4.1.0) and Monocle (version 2). Cells with more than 2000 or less than 200 genes detected or more than 10% mitochondrial gene expression were first filtered out as low-quality cells. Principal component analysis (PCA) was performed on the variable genes, and 20 principal components were used for cell clustering (resolution = 0.15) and uniform manifold approximation and projection (UMAP) dimensional reduction. The cluster markers were found using the FindAllMarkers function. Monocle 2, which employs a machine-learning algorithm known as reverse graph embedding (RGE), was used to construct differentiation trajectories from scRNA-seq data. The top 150 marker genes for the three clusters were used to construct the trajectory. Module scores were calculated using the AddModuleScore function with default parameters. The large data sets have been deposited to Sequence Read Archive (SRA) database with access numbers SAMN30526153, SAMN30526154, SAMN30526155 for scRNA sequencing and SAMN30526295, SAMN30526296, SAMN30526297 for bulk RNA sequencing.

Statistics. Data presentation and statistical analyses were carried out using Prism 8 software (GraphPad) and graphed as mean ± standard error of the mean. For cGVHD clinical scores, all animals were examined for GVHD clinical signs on day 0 as a reference point, and non-reference point clinical score data were analyzed using mixed model tests to determine any statistical significance between groups ($p < .05$). Murine survival data of GVHD was analyzed with a log-rank (Mantel-Cox) test to determine any statistical significance between groups ($p < .05$). Fisher's exact test was performed on day 60 of the xenograft model to determine difference in survival between groups. In BALB/c model of acute GVHD, clinical scoring in this model of aGVHD data was treated as 'last observation carried forward' (LOCF) for any mouse in any group that succumbed to mortality prior to experimental endpoint (day 80 post-

BMT). ROUT method calculated using Prism 8 software was designed to detect any significant statistical outliers prior to initiating the study, and four outlier values were found with this method (Figure 1D: *Fli1*^{WT} n=2, *Fli1*$^{fl/wt}$Cre+ n=1; Figure 2D (IL-17A): *Fli1*^{WT} n=1), inclusion or removal of the outliers did not impact the conclusions of these data. For all other data, differences between only two experimental groups were compared using an unpaired 2-tailed Student *t* test to determine any statistical significance ($p < .05$); When comparing >2 groups, a one-way analysis of variance (ANOVA) using Tukey or Bonferroni correction for multiple comparisons was performed to determine any statistical significance ($p < .05$).

Study approval. All strains were maintained in a specific pathogen-free facility at an American Association for Laboratory Animal Care–accredited Animal Resource Center the Medical University of South Carolina (MUSC, Charleston, SC) and Medical College of Wisconsin (MCW, Milwaukee, WI). Animal experiments were approved by the MUSC Institutional Animal Care and Use Committee and MCW Animal Use Application (AUA).

Supplementary References

1. Schutt SD, Wu Y, Tang C-HA, Bastian D, Nguyen H, Sofi MH, et al. Inhibition of the IRE-1α/XBP-1 pathway prevents chronic GVHD and preserves the GVL effect in mice. *Blood Advances.* 2018;2(4):414-27.
2. Schutt SD, Fu J, Nguyen H, Bastian D, Heinrichs J, Wu Y, et al. Inhibition of BTK and ITK with Ibrutinib Is Effective in the Prevention of Chronic Graft-versus-Host Disease in Mice. *PloS one.* 2015;10(9):e0137641.
3. Heinrichs J, Li J, Nguyen H, Wu Y, Bastian D, Daethanasanmak A, et al. CD8(+) Tregs promote GVHD prevention and overcome the impaired GVL effect mediated by CD4(+) Tregs in mice. *Oncoimmunology.* 2016;5(6):e1146842.
4. Sofi MH, Heinrichs J, Dany M, Nguyen H, Dai M, Bastian D, et al. Ceramide synthesis regulates T cell activity and GVHD development. *JCI insight.* 2017;2(10).
5. Pan W, Zhu S, Dai D, Tang Y, Yao Y, and Shen N. T Cell Transfer Model of Colitis. *Bio-Protocol.* 2016;6(13).
6. Li YJ, Zhao X, Vecchiarelli-Federico LM, Li Y, Datti A, Cheng Y, et al. Drug-mediated inhibition of Fli-1 for the treatment of leukemia. *Blood cancer journal.* 2012;2(1):e54.
7. Nitiss JL, Soans E, Rogojina A, Seth A, and Mishina M. Topoisomerase assays. *Curr Protoc Pharmacol.* 2012;Chapter 3:Unit 3
